# Global trends and emerging themes in youth physical literacy research: a bibliometric analysis (2000–2025)

**DOI:** 10.3389/fspor.2026.1859748

**Published:** 2026-07-22

**Authors:** Zhao Zishan, Asha Hasnimy Mohd Hashim

**Affiliations:** Faculty of Educational Sciences and Technology, Universiti Teknologi Malaysia, Johor Bahru, Malaysia

**Keywords:** bibliometric analysis, motor competence, physical activity, physical education, physical literacy, science mapping, youth

## Abstract

**Background:**

Physical literacy (PL) has emerged as an important framework for understanding children's and adolescents' motivation, confidence, physical competence, knowledge, and engagement in lifelong physical activity. Although youth physical literacy research has expanded rapidly, limited bibliometric evidence has systematically mapped its global development, intellectual structure, and emerging thematic trends. This study aimed to examine the evolution of youth physical literacy research from 2000 to 2025 and identify major research themes, collaboration patterns, and emerging directions.

**Materials and methods:**

A bibliometric analysis was conducted using records retrieved from the Web of Science Core Collection and Scopus databases within a multi-database validation framework. After data cleaning, deduplication, and exclusion of records outside the study period, 856 publications published between 2000 and 2025 were included. Bibliometrix, VOSviewer, and CiteSpace were used to examine publication trends, collaboration networks, influential contributors, keyword co-occurrence patterns, thematic clusters, timeline evolution, and burst keywords.

**Results:**

Youth physical literacy research showed a sustained increase in publications, particularly after 2015. Canada, Australia, China, the United States, and the United Kingdom were among the most productive countries. Keyword and cluster analyses identified major themes related to physical education, motor competence, physical activity, physical competence, assessment and validation, fitness, health literacy, and public health. Timeline and burst analyses further suggested increasing attention to psychological, educational, and well-being-related themes within the youth physical literacy literature. Cross-database comparison showed consistent patterns in publication trends, leading contributors, and core thematic structures, supporting the robustness of the identified research patterns.

**Conclusion:**

This study provides a systematic and validated mapping of the global research landscape of youth physical literacy from 2000 to 2025. The findings suggest that youth physical literacy research has expanded from physical education, motor competence, and assessment-oriented topics toward broader educational, psychosocial, and health-related themes. These results offer a knowledge map for researchers, educators, and policymakers interested in advancing youth physical literacy in educational contexts and supporting holistic student development.

## 1 Introduction

Physical activity has long been recognized as an important contributor to physical and psychological development among children and adolescents, particularly within educational settings (1). In recent years, increasing scholarly attention has shifted toward the concept of physical literacy, which extends beyond physical competence to encompass motivation, confidence, knowledge, and engagement in lifelong physical activity (2–5). As a multidimensional construct, physical literacy has been increasingly linked to a range of developmental outcomes among youth, including health promotion, physical activity participation, movement competence, and psychosocial development (6–8).

In this study, student well-being is used in a broad interpretive sense to refer to psychological, social, and educational dimensions appearing in the physical literacy literature, including mental health, motivation, self-concept, engagement, and social functioning. It was not used as a direct search filter, but was considered as an emergent theme when interpreting keyword, cluster, timeline, and burst analyses.

Alongside this growing interest, the promotion of positive developmental and well-being-related outcomes through physical activity has become an important concern in both educational research and policy discourse. School environments provide structured and socially embedded contexts in which physical literacy can be developed and expressed, making them particularly relevant for understanding how physical activity contributes to broader developmental processes (9, 10). Emerging evidence suggests that physical literacy is associated not only with physical health indicators but also with psychosocial outcomes such as emotional well-being, interpersonal skills, and adaptive functioning in academic settings (11, 12). Consistent with this perspective, a meta-analysis reported that physical activity-based interventions exert significant positive effects on children's and adolescents' physical self-concept, which may further enhance global self-worth (4).

Despite the rapid expansion of research in this area, the literature remains fragmented, and there is still limited synthesis of its global development, intellectual structure, and emerging thematic directions (13, 14). Previous bibliometric studies have made important contributions by mapping the broader development of physical literacy research and identifying major trends in the field (15, 16). However, these studies have generally focused on the overall physical literacy literature or specific health-related dimensions, with comparatively limited attention to youth-oriented physical literacy research across multiple databases and to how educational, psychosocial, and well-being-related themes emerge within this body of literature. As a result, it remains unclear how this field has evolved over time and how its knowledge structure aligns with broader educational and well-being-oriented agendas. In addition, limited evidence is available regarding whether the major bibliometric patterns identified in previous single-database studies remain consistent across multiple bibliographic sources.

To address this gap, the present study conducts a bibliometric analysis of global youth physical literacy research from 2000 to 2025 using data retrieved from the Web of Science Core Collection and Scopus databases. Specifically, this study examines publication trends, leading countries and institutions, author collaboration networks, keyword co-occurrence patterns, thematic clusters, timeline evolution, and burst keywords. Beyond descriptive mapping, the findings are interpreted in relation to school-based physical activity and student well-being as emerging themes identified through keyword co-occurrence, clustering, timeline, and burst analyses within the youth physical literacy literature. This approach enables the study to clarify how the thematic focus of youth physical literacy research has shifted from a traditional emphasis on motor competence and physical education toward broader psychological, social, behavioral, and health-related dimensions. In doing so, the study provides a structured knowledge map for researchers, educators, and policymakers interested in advancing youth physical literacy and holistic student development.

## 2 Materials and methods

### 2.1 Research design and data sources

This study employed a bibliometric analysis and scientific knowledge mapping design to examine the global development, collaboration patterns, intellectual structure, and emerging themes of youth physical literacy research. Bibliometric analysis was considered appropriate because it enables the systematic mapping of publication trends, influential contributors, thematic structures, and temporal evolution within a research field (17, 18).

To enhance data coverage and reduce potential database-specific bias, bibliographic records were retrieved from two major international databases: the Web of Science Core Collection (WoSCC) and Scopus. WoSCC, including the Science Citation Index Expanded (SCI-Expanded) and Social Sciences Citation Index (SSCI), was selected because of its standardized citation metadata, high compatibility with citation-based mapping tools, and frequent use in bibliometric studies. Scopus was included because of its broad journal coverage and comparatively rich keyword metadata, which are useful for thematic and co-occurrence analyses. The complementary roles of the two databases are summarized in Table 1.

**Table 1:** Comparative characteristics of WoSCC and scopus and their analytical roles.

Aspect	WoSCC	Scopus	Role in this study
Coverage focus	High-impact journals; strong citation indexing	Broad multidisciplinary journal coverage	Complementary source coverage
Citation metadata	Highly standardized; strong cited-reference structure	Good metadata; broader source diversity	Used for citation-based mapping and descriptive expansion
Affiliation consistency	Strong institutional standardization	Moderate variation across records	WoSCC prioritized for collaboration networks
Keyword completeness	Moderate	Stronger author keyword coverage	Scopus prioritized for keyword co-occurrence analysis
CiteSpace compatibility	Excellent (plain text format)	Limited direct compatibility	WoSCC used for CiteSpace analyses
Primary analytical applications	Collaboration networks, clustering, timeline, burst detection	Keyword mapping and thematic enrichment	Merged dataset used for descriptive and performance analyses

### 2.2 Search strategy and eligibility criteria

A systematic search strategy was applied in both databases using equivalent keyword combinations adapted to their respective search fields. In WoSCC, the Topic (TS) field was used, whereas in Scopus, the TITLE-ABS-KEY field was used. The search focused on the term “physical literacy” in combination with youth-related terms, including youth, adolescent*, student*, “young people”, child*, school*, college*, and university*.

The WoSCC search string was: TS = (“physical literacy”) AND TS = (youth OR adolescent* OR student* OR “young people” OR child* OR school* OR college* OR university*). The Scopus search string was: TITLE-ABS-KEY(“physical literacy”) AND TITLE-ABS-KEY(youth OR adolescent* OR student* OR “young people” OR child* OR school* OR college* OR university*).

The search was limited to English-language articles and review papers published between 2000 and 2025. Records were retrieved in April 2026 and exported with full bibliographic information and cited references where available.

**Figure 1:**
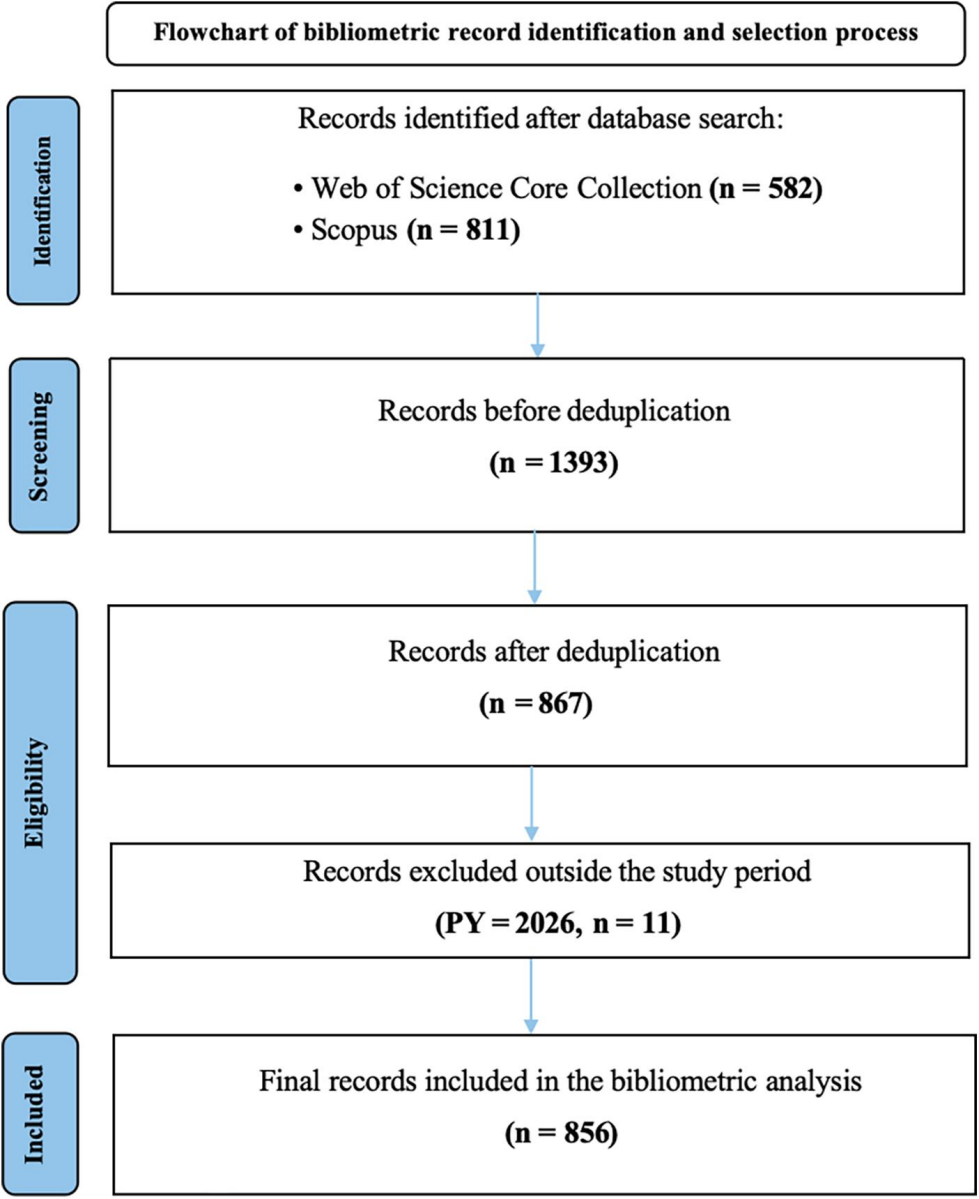
Flowchart of bibliometric record identification, merging, deduplication, and final inclusion based on the WoSCC and Scopus databases.

Records were included if they met the following criteria: (1) focused on physical literacy; (2) involved youth-related populations or educational contexts as indicated by the search terms; (3) were published as articles or reviews; (4) were written in English; and (5) fell within the predefined study period of 2000–2025. Records that did not meet these criteria or lacked sufficient bibliographic information for bibliometric analysis were excluded. The identification, merging, and deduplication procedures are shown in Figure 1. The flowchart represents a bibliometric identification and deduplication process rather than a PRISMA-based systematic review screening process.

### 2.3 Data export, deduplication, and cleaning

The initial search yielded 582 records from WoSCC and 811 records from Scopus. All records were exported in their original database formats and then merged for further processing. Deduplication was conducted using the Bibliometrix package in R, primarily through DOI matching and title matching. Records without DOI information were checked through title comparison and manual verification where necessary. To further clarify the data-processing procedure, duplicate records were identified based on combinations of DOI, title, and bibliographic information.

During data cleaning and verification, 11 records with a publication year of 2026 were identified and excluded to ensure consistency with the predefined study period. Therefore, the final dataset included 856 publications published between 2000 and 2025.

Before analysis, bibliographic metadata were cleaned and standardized to improve consistency across databases. Author names, institutional names, country names, and keywords were reviewed for inconsistencies caused by spelling variations, abbreviations, alternative naming forms, or singular/plural variants. For example, equivalent institutional names appearing in different languages or formats were merged into a single standardized form. Keywords with equivalent meanings or singular/plural variants were also standardized where necessary to reduce fragmentation in keyword co-occurrence analysis.

Country-level analyses were based on author affiliations using full counting, whereby publications with multiple country affiliations contributed to each participating country. The same counting logic was applied consistently throughout the study. These procedures were conducted to improve the reliability of institutional, country-level, and thematic analyses.

During the merging and deduplication process, duplicate records indexed in both WoSCC and Scopus were consolidated into a single record to avoid double counting. Consequently, country-level affiliation frequencies in the merged dataset should not be interpreted as the sum of frequencies reported by the two databases. In some cases, country frequencies in the merged dataset may be lower than those observed in an individual database because duplicate records were consolidated during harmonization and deduplication.

Duplicate records were identified primarily through DOI and title matching using the Bibliometrix merging procedure. When a publication was indexed in both WoSCC and Scopus, a single consolidated record was retained in the merged dataset, generally preserving the WoSCC metadata structure, while Scopus contributed additional unique records not indexed in WoSCC. Country and institutional names were subsequently standardized to improve consistency across records.

Because WoSCC and Scopus differ in affiliation metadata structure and coverage, country-level frequencies in the merged dataset reflect the final deduplicated records rather than the sum of database-specific affiliation counts. Therefore, merged country frequencies may be slightly lower than those reported in individual databases.

### 2.4 Bibliometric analysis

Bibliometric analyses were conducted using Bibliometrix (v5.4.1) and its Biblioshiny interface (17). The merged WoSCC–Scopus dataset was used for descriptive and performance-based analyses, including annual publication trends, source productivity, leading authors, countries, institutions, citation indicators, and three-field plot visualization. Key indicators included number of publications (NP), total citations (TC), average citations, and h-index.

Because the study used a multi-database dataset, a mixed-source analytical strategy was adopted according to software compatibility and metadata completeness (18). The merged dataset was used for overall descriptive and performance indicators. Scopus data were used primarily for keyword co-occurrence analysis because of their comparatively complete keyword metadata. WoSCC data were used for CiteSpace-based analyses because CiteSpace performs most reliably with standardized WoSCC plain-text records.

### 2.5 Visualization and network parameters

VOSviewer (v1.6.20) was used to construct keyword co-occurrence networks and selected collaboration maps (19). For VOSviewer analyses, full counting and association-strength normalization were adopted. For keyword co-occurrence analysis, a minimum occurrence threshold of 10 was applied. In addition, thesaurus-based keyword cleaning was conducted to merge synonymous terms and remove non-informative keywords in order to improve the interpretability of the network.

CiteSpace (v6.4.R1) was used to examine temporal and intellectual structures, including keyword clustering, timeline visualization, and burst keyword detection. CiteSpace analyses were conducted using WoSCC plain-text records (20, 21). For CiteSpace analyses, one-year time slicing was applied, the g-index was selected with k = 25, and no pruning was applied. Burst detection was performed using CiteSpace's implementation of the Kleinberg algorithm with a two-state model, γ = 0.6, and a minimum burst duration of 2 years. The top 15 burst keywords were reported. The analysis was restricted to records within the 2000–2025 study period. The clustering solution yielded a modularity Q value of 0.4114 and a weighted mean silhouette value of 0.7478. The modularity value indicates a moderately structured clustering network, while the silhouette value suggests satisfactory cluster homogeneity. Therefore, the clustering solution was considered reasonably structured and suitable for thematic interpretation. The datasets, software packages, and main analytical settings used for each analysis are summarized in Table 2.

**Table 2:** Databases, software, and analytical settings used in the bibliometric analysis.

Analysis	Database	Software	Main settings
Publication trends	Merged WoSCC–Scopus	Bibliometrix	Annual publication counts and growth trends
Country productivity	Merged WoSCC–Scopus	Bibliometrix	NP, TC, average citations
Institution productivity	Merged WoSCC–Scopus	Bibliometrix	NP, TC, h-index
Author productivity	Merged WoSCC–Scopus	Bibliometrix	NP, TC, h-index
Three-field plot	Merged WoSCC–Scopus	Bibliometrix	Relationships among institutions, authors, and countries
Keyword co-occurrence	Scopus	VOSviewer	Full counting; association-strength normalization; minimum occurrence threshold = 10; thesaurus-based keyword cleaning applied
Author density visualization	Scopus	VOSviewer	Full counting; association-strength normalization; density visualization of influential authors
Country collaboration network	WoSCC	CiteSpace	Time slicing = 1 year; g-index (k = 25); LRF = 2.5; L/N = 10; LBY = 5; e = 1.0; pruning = none
Institution collaboration network	WoSCC	CiteSpace	Time slicing = 1 year; g-index (k = 25); LRF = 2.5; L/N = 10; LBY = 5; e = 1.0; pruning = none
Author collaboration network	WoSCC	CiteSpace	Time slicing = 1 year; g-index (k = 25); LRF = 2.5; L/N = 10; LBY = 5; e = 1.0; pruning = none
Keyword clustering	WoSCC	CiteSpace	Time slicing = 1 year; g-index (k = 25); LRF = 2.5; L/N = 10; LBY = 5; e = 1.0; pruning = none; Q = 0.4114; S = 0.7478
Timeline analysis	WoSCC	CiteSpace	Time slicing = 1 year; g-index (k = 25); LRF = 2.5; L/N = 10; LBY = 5; e = 1.0; pruning = none
Burst detection	WoSCC	CiteSpace	Kleinberg burst-detection algorithm; *γ* = 0.6; minimum duration = 2 years; number of states = 2; top 15 burst keywords reported

### 2.6 Cross-database validation

To improve methodological robustness, this study employed a multi-database validation strategy using WoSCC and Scopus. Equivalent search strategies were independently applied in both databases, with syntax adapted to each platform. Prior to merging, selected core indicators were compared between WoSCC and Scopus, including annual publication trends, leading contributing countries, and high-frequency keyword profiles.

The purpose of this comparison was not to treat the two databases as identical, but to assess whether the major bibliometric patterns were consistent across independent sources. The observed convergence across publication trends, leading contributors, and core themes supported the robustness of the identified research patterns. Accordingly, the combined use of WoSCC and Scopus functioned as both a source-expansion strategy and a cross-validation framework, strengthening the reliability of the present findings. Minor discrepancies between the WoSCC and Scopus datasets were expected because of differences in journal coverage, indexing policies, and metadata processing. However, the consistency of the overall publication trends, leading contributors, and thematic patterns supports the robustness of the findings.

## 3 Results

### 3.1 General characteristics of the retrieved literature

The final merged dataset comprised 856 publications on youth physical literacy after merging and deduplicating records retrieved from the Web of Science Core Collection (WoSCC) and Scopus databases. Although the search period was defined as 2000–2025, no eligible publications were identified before 2007. Therefore, the effective publication period analyzed in this study was 2007–2025.

The retrieved publications were distributed across 257 publication sources, indicating broad disciplinary coverage of youth physical literacy research. A total of 2,493 authors contributed to the literature, reflecting substantial scholarly participation in the field. Only 45 documents were single-authored, suggesting that collaborative authorship has become the dominant pattern of knowledge production.

The average number of co-authors per document was 5.25, indicating a relatively high level of research collaboration. International co-authorship accounted for 26.05% of publications, demonstrating the growing importance of cross-national cooperation in advancing youth physical literacy research.

The mean document age was 4.12 years, suggesting that the field remains relatively young and continues to develop rapidly. The average number of citations per document was 15.45, indicating a moderate level of scholarly influence. In addition, a total of 1,644 author keywords were identified, highlighting considerable thematic diversity and the evolving conceptual structure of the field.

### 3.2 Cross-database validation of key bibliometric indicators

The robustness of the bibliometric findings was evaluated through an independent comparison of key indicators derived from the WoSCC and Scopus databases before data merging. The comparative results are presented in Table 3.

**Table 3:** Cross-database validation of key bibliometric indicators in the WoSCC and scopus datasets before data merging.

Indicator	WoSCC	Scopus	Interpretation
Annual publication trend	Limited output before 2010; steady growth after 2015; rapid expansion after 2020	Limited output before 2010; steady growth after 2015; rapid expansion after 2020	Highly consistent temporal evolution
Top productive country	Canada (478)	Canada (577)	Consistent leader
2nd country	China (273)	USA (348)	Minor rank variation
3rd country	Australia (271)	Australia (282)	Shared core country
4th country	USA (228)	UK (206)	Shared core country
5th country	UK (167)	China (194)	Shared core country
Shared top-5 countries	Canada, China, Australia, USA, UK	Canada, USA, Australia, UK, China	Full overlap
Top keyword	Physical literacy	Physical literacy	Exact match
Other core keywords	Physical activity; Fundamental movement skills; Adolescents; Intervention; Motor competence; Motor development; Mental health; Early childhood; Physical education	Physical activity; Motivation; Adolescents; Fundamental movement skills; Intervention; Physical fitness; Physical competence; Early childhood; Motor competence	Strong thematic overlap

Rankings, publication trends, country frequencies, and keyword profiles were generated independently from the WoSCC and Scopus datasets using Biblioshiny before data merging. Country frequencies were based on author affiliations. Because publications with multiple country affiliations may be counted for each contributing country, summed country frequencies may exceed the total number of publications. Keyword comparison was based on high-frequency and co-occurrence keywords identified separately in each database.

Both databases demonstrated highly similar publication trajectories. Research output remained limited during the early years of the study period, increased steadily after 2015, and expanded rapidly after 2020. This consistent temporal pattern indicates that the overall growth of youth physical literacy research was not dependent on a single database source.

At the country level, both databases identified Canada, Australia, China, the United States, and the United Kingdom as the dominant contributors to the field, although minor differences were observed in their ranking positions. Canada ranked first in both datasets, while the remaining four countries appeared consistently among the top contributors. The complete overlap of the top five countries suggests strong geographical consistency across databases.

At the thematic level, physical literacy emerged as the most central keyword in both datasets. Furthermore, substantial overlap was observed among the major research themes, including physical activity, adolescents, intervention, motor competence, and fundamental movement skills. While some database-specific variations were present, both datasets highlighted similar educational, developmental, and health-related dimensions of youth physical literacy research.

Overall, the strong agreement between WoSCC and Scopus in publication trends, leading countries, and keyword structures supports the robustness of the merged dataset. These findings suggest that the major research patterns identified in the present study are robust and unlikely to be artifacts of database-specific coverage.

### 3.3 Annual scientific production

**Figure 2:**
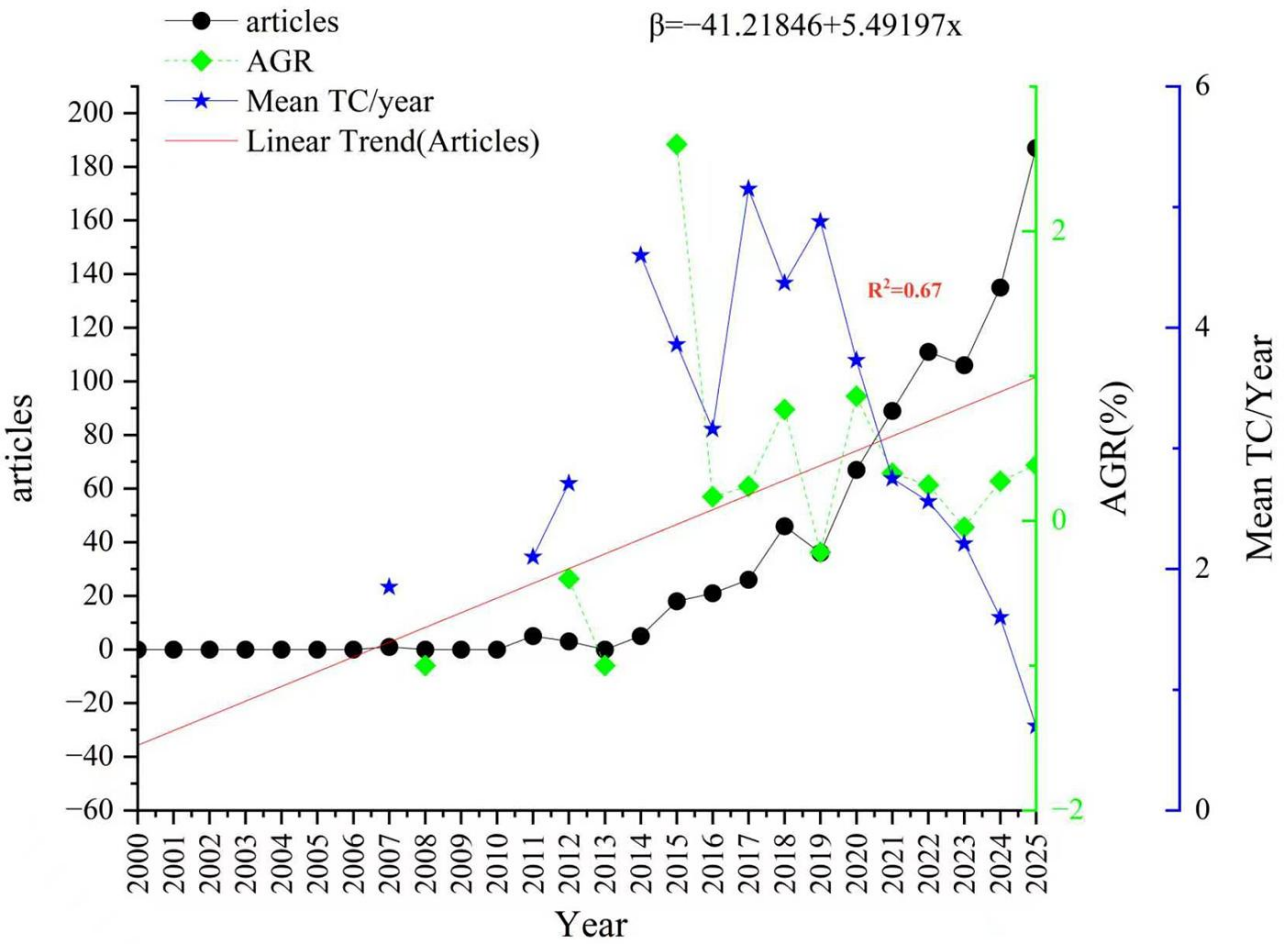
Annual scientific production and citation trends in youth physical literacy research based on the merged WoSCC–Scopus dataset.

Figure 2 illustrates the annual scientific production, annual growth rate (AGR), and mean citations per year in youth physical literacy research from 2000 to 2025. Overall, annual scientific production showed a clear upward trajectory throughout the study period. Although the search period covered 2000–2025, publications first appeared in 2007, indicating that youth physical literacy emerged relatively recently as an independent research field. During the initial stage (2007–2014), research output remained limited, with only sporadic publications and several years recording no publications.

Research productivity increased substantially after 2015, marking the beginning of a sustained growth phase. Annual publications increased from 18 in 2015 to 67 in 2020 and continued to rise thereafter, reaching 89 publications in 2021, 111 in 2022, 106 in 2023, 135 in 2024, and a peak of 187 publications in 2025. Linear regression analysis further confirmed a significant upward trend in publication output over time (β = 5.49, R^2^ = 0.67), indicating continuously increasing scholarly attention to youth physical literacy.

The annual growth rate (AGR) exhibited considerable fluctuations throughout the study period. Following several years of limited or negative growth, publication output increased sharply in 2015 (AGR = 260.0%). Growth remained positive in most subsequent years, although temporary declines were observed in 2019 (−21.74%) and 2023 (−4.50%). After these short-term decreases, publication output recovered and continued to expand, with AGR reaching 27.36% in 2024 and 38.52% in 2025. Overall, despite periodic fluctuations, the long-term trajectory demonstrates sustained expansion of the research field.

Mean citations per year followed a different pattern from publication output. Citation impact gradually increased during the early years of the field and reached its highest level in 2017 (5.15 citations per year). Thereafter, the average citation rate declined steadily, decreasing from 4.88 in 2019 to 3.73 in 2020, 2.75 in 2021, 2.56 in 2022, 2.21 in 2023, 1.60 in 2024, and 0.70 in 2025. This declining trend is expected, as recently published articles have had substantially less time to accumulate citations. Taken together, these findings reflect the rapid expansion of youth physical literacy research while also illustrating the expected citation lag associated with recently published studies.

### 3.4 Scientific contributions by country

At the country level, substantial variation was observed in scientific production, citation impact, and international collaboration patterns within youth physical literacy research (Table 4).

**Table 4:** Scientific production, citation impact, and corresponding-author collaboration by country in youth physical literacy research.

Scientific production by country		Most cited countries			Corresponding authors' countries			
Country	Freq	Country	TC	Average article citations	Country	Articles	Articles (%)	MCP (%)
Canada	521	Canada	3,987	23.9	Canada	167	19.5	16.2
Australia	294	UK	1,832	28.6	China	110	12.9	31.8
China	283	USA	1,594	16.1	USA	99	11.6	19.2
USA	281	Australia	1,333	20.5	Australia	65	7.6	43.1
UK	184	China	1,087	9.9	UK	64	7.5	26.6
Spain	121	Germany	533	24.2	Spain	25	2.9	40.0
Chile	64	Portugal	262	18.7	Indonesia	23	2.7	0.0
Denmark	64	Denmark	194	17.6	Germany	22	2.6	18.2
Germany	50	Spain	192	7.7	Croatia	15	1.8	26.7
Portugal	44	Italy	191	15.9	Chile	14	1.6	71.4

Descriptive statistics were generated from the merged WoSCC–Scopus dataset after deduplication. Freq refers to publication frequency based on author affiliations using full counting; therefore, publications with authors from multiple countries may contribute to more than one country. TC and average article citations are based on corresponding-author country records generated by Bibliometrix. Average article citations were calculated as TC divided by the number of corresponding-author articles, rather than by Freq. MCP=multiple-country publications, referring to the percentage of corresponding-author publications involving international collaboration.

Canada was the most productive country, contributing 521 publications, followed by Australia (294), China (283), the United States (281), and the United Kingdom (184). These findings suggest that research activity was concentrated within a relatively small group of countries.

Regarding citation impact, Canada accumulated the highest number of total citations (TC = 3,987), followed by the United Kingdom (TC = 1,832), the United States (TC = 1,594), Australia (TC = 1,333), and China (TC = 1,087). However, average article citations showed a somewhat different pattern. The United Kingdom demonstrated the highest average citation impact (28.6 citations per article), followed by Germany (24.2), Canada (23.9), and Australia (20.5). These findings suggest that publication quantity and citation influence were not always aligned across countries.

Analysis of corresponding authors' countries revealed that Canada accounted for the largest share of corresponding-author publications (Articles = 167, 19.5%), followed by China (110, 12.9%) and the United States (99, 11.6%). In terms of international collaboration, Chile exhibited the highest proportion of multiple-country publications (MCP = 71.4%), followed by Australia (43.1%) and Spain (40.0%). In contrast, Canada showed a relatively lower MCP ratio (16.2%), indicating that a substantial proportion of its research output was produced through domestic collaborations.

**Figure 3:**
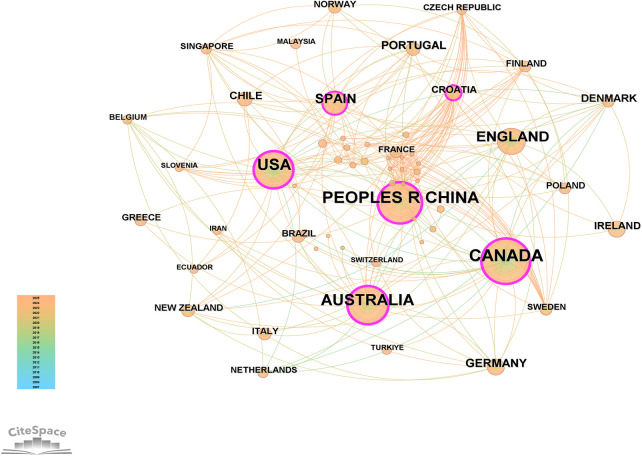
International collaboration network among countries in youth physical literacy research based on the WoSCC dataset.

Figure 3 presents the international collaboration network among countries involved in youth physical literacy research. Canada maintained extensive collaborative relationships with several major contributing countries and occupied a central position within the global collaboration network. Australia, China, the United States, and the United Kingdom also played important roles in international collaboration, reflecting their substantial contributions to global knowledge production in youth physical literacy research.

In addition, several European countries, including England, Spain, Portugal, Germany, Denmark, and Ireland, displayed dense collaborative connections, highlighting strong regional cooperation within Europe. Notably, Spain and Croatia occupied relatively important bridging positions within the collaboration network.

Overall, youth physical literacy research is concentrated within a relatively small group of highly productive countries while simultaneously demonstrating increasing levels of international cooperation. The collaboration network suggests that knowledge production in this field has become progressively more globalized and interconnected.

### 3.5 Institutional contributions

**Figure 4:**
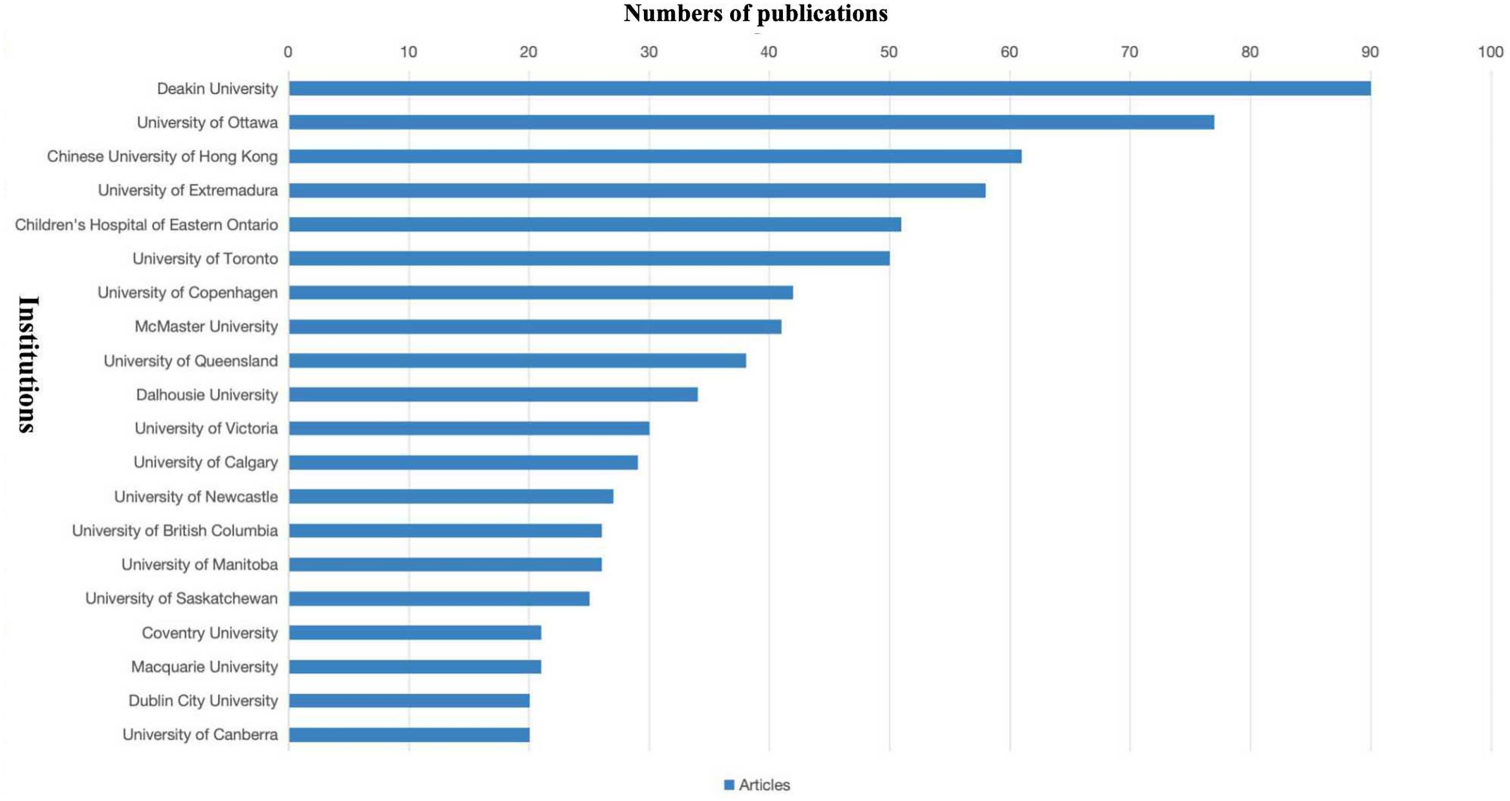
Leading institutions in youth physical literacy research based on the merged WoSCC–scopus dataset.

Research output at the institutional level was concentrated among a relatively small group of universities and research organizations (Figure 4). Deakin University was the most productive institution with 90 publications, followed by the University of Ottawa (77), the Chinese University of Hong Kong (61), and the University of Extremadura (58). Other leading contributors included the Children's Hospital of Eastern Ontario (51), the University of Toronto (50), the University of Copenhagen (42), and McMaster University (41). Overall, the most productive institutions were primarily located in Australia, Canada, Hong Kong SAR/China, and Europe, indicating that research output was concentrated within several well-established research centers.

**Figure 5:**
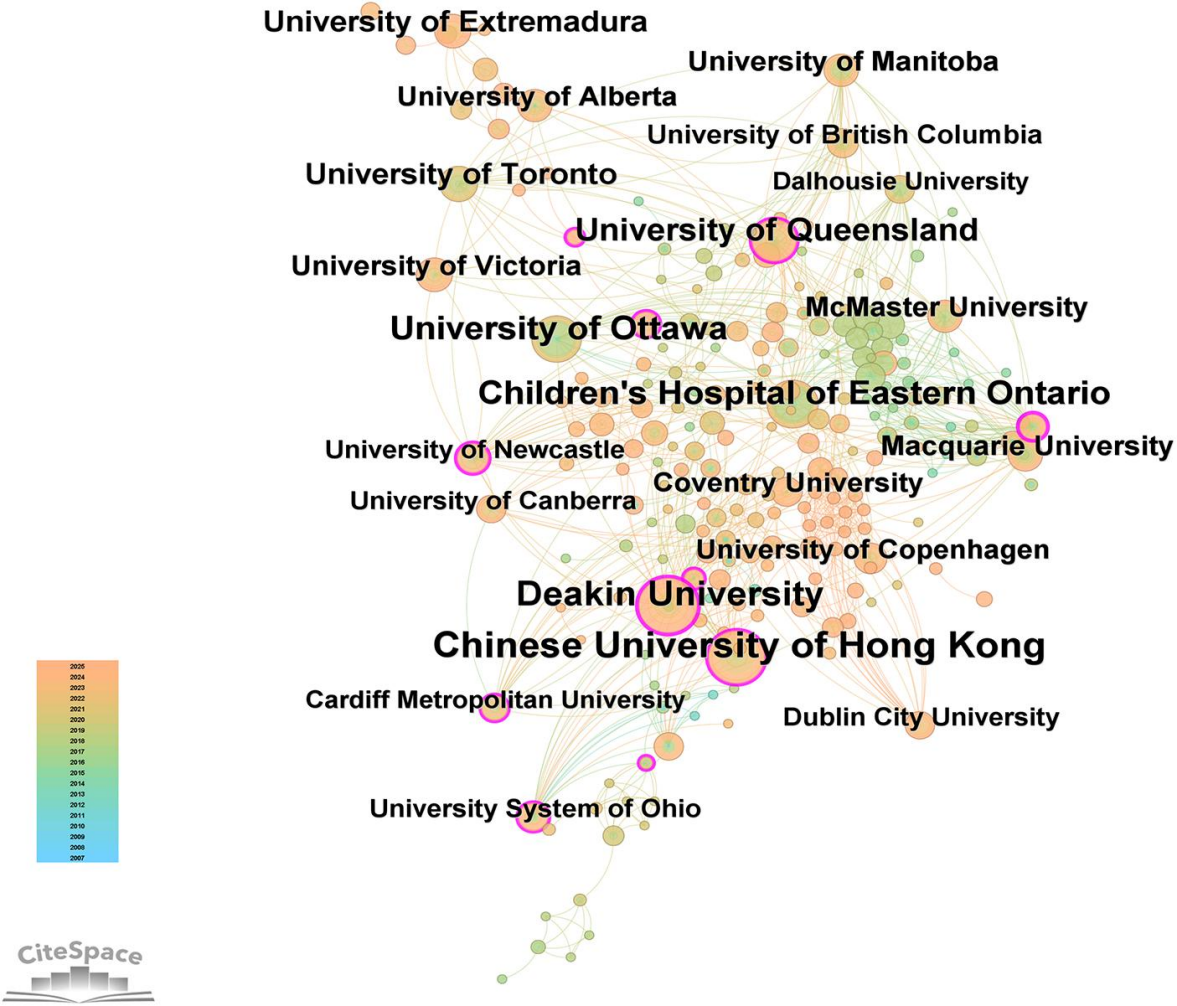
Institutional collaboration network in youth physical literacy research based on the WoSCC dataset.

Figure 5 presents the institutional collaboration network generated from the WoSCC dataset, revealing extensive collaborative relationships among leading research institutions. Deakin University, the University of Ottawa, the Chinese University of Hong Kong, and the Children's Hospital of Eastern Ontario occupied central positions within the network and maintained extensive collaborative relationships with other institutions. The University of Toronto, McMaster University, the University of Queensland, and the University of Copenhagen also maintained active collaborative relationships with other institutions.

The collaboration network demonstrates substantial international cooperation among institutions, particularly those located in Canada, Australia, Europe, and Hong Kong SAR/China. Several institutions, including the University of Extremadura, Macquarie University, the University of Newcastle, and the University of Victoria, also maintained active collaborative relationships with multiple partners, contributing to the overall connectivity of the network.

Overall, youth physical literacy research exhibits a highly interconnected institutional collaboration network, with a relatively small number of leading institutions playing central roles in both research productivity and collaborative activities.

### 3.6 Journal analysis

Based on the merged WoSCC–Scopus dataset, publications on youth physical literacy were distributed across journals from multiple disciplines, including public health, physical education, sport science, psychology, and multidisciplinary research (Table 5). In terms of publication output, the *International Journal of Environmental Research and Public Health* ranked first with 42 articles, followed by *BMC Public Health* (41), *Children (Basel)* (34), the *Journal of Teaching in Physical Education* (31), and *Frontiers in Sports and Active Living* (24). These findings suggest that youth physical literacy research is disseminated across both health-oriented and physical education-related journals.

**Table 5:** Leading journals in youth physical literacy research: publication output, local citation impact, and temporal distribution.

Journals	Articles	Local TC	h-index	PY_start
International Journal of Environmental Research and Public Health	42	803	17	2,016
BMC Public Health	41	1,383	20	2,014
Children (Basel)	34	280	10	2018
Journal of Teaching in Physical Education	31	651	15	2016
Frontiers in Sports and Active Living	24	125	6	2022
Physical Education and Sport Pedagogy	23	360	13	2015
PLOS ONE	22	310	8	2017
Frontiers in Psychology	21	314	8	2020
Frontiers in Public Health	21	240	9	2019
Journal of Physical Education, Recreation and Dance	15	86	4	2018
Sport, Education and Society	14	211	8	2007
European Physical Education Review	13	309	9	2011
Education Sciences	12	40	3	2021
Applied Physiology, Nutrition, and Metabolism	11	152	7	2019
Journal of Exercise Science & Fitness	11	239	8	2018
Journal of Physical Education and Sport	11	58	4	2016
Journal of Sports Sciences	11	217	8	2016
Journal of Sport and Health Science	10	655	10	2015
Research Quarterly for Exercise and Sport	10	174	5	2016
Retos	10	29	3	2017

Articles = number of publications; Local TC=local total citations within the merged WoSCC–Scopus dataset; h-index = Hirsch index; PY_start = first year in which the journal appeared in the dataset. Journal titles were standardized across the merged WoSCC–Scopus dataset before analysis.

Regarding local citation impact, *BMC Public Health* recorded the highest local total citations (Local TC = 1,383) and the highest h-index (20), despite ranking second in publication output. The *International Journal of Environmental Research and Public Health* ranked second in local citation impact (Local TC = 803; h-index = 17). Several journals with comparatively fewer publications also showed notable local citation influence. For example, the *Journal of Sport and Health Science* accumulated 655 local citations from only 10 articles, while the *Journal of Teaching in Physical Education* received 651 local citations from 31 articles. Similarly, *Physical Education and Sport Pedagogy* and *European Physical Education Review* also showed relatively strong local citation performance.

The temporal distribution of journals also reflects the development of publication outlets in this field. *Sport, Education and Society* was the earliest journal represented in the dataset (PY_start = 2007), followed by *European Physical Education Review* (2011), *BMC Public Health* (2014), and *Physical Education and Sport Pedagogy* and the *Journal of Sport and Health Science* (both 2015). Since 2016, youth physical literacy research has appeared in a wider range of journals, including the *International Journal of Environmental Research* and *Public Health*, the *Journal of Teaching in Physical Education*, the *Journal of Physical Education and Sport*, the *Journal of Sports Sciences, and Research Quarterly for Exercise and Sport*. More recent outlets, such as *Frontiers in Psychology* (2020), *Education Sciences* (2021), and *Frontiers in Sports and Active Living* (2022), further indicate the continued expansion of publication venues.

Overall, the journal landscape shows both concentration in several leading outlets and dissemination across a broad range of disciplinary areas. These findings suggest that youth physical literacy research is not confined to a single academic field but is increasingly represented across public health, education, sport science, psychology, and multidisciplinary journals.

### 3.7 Author-level contributions

#### 3.7.1 Leading authors: productivity and academic impact

**Figure 6:**
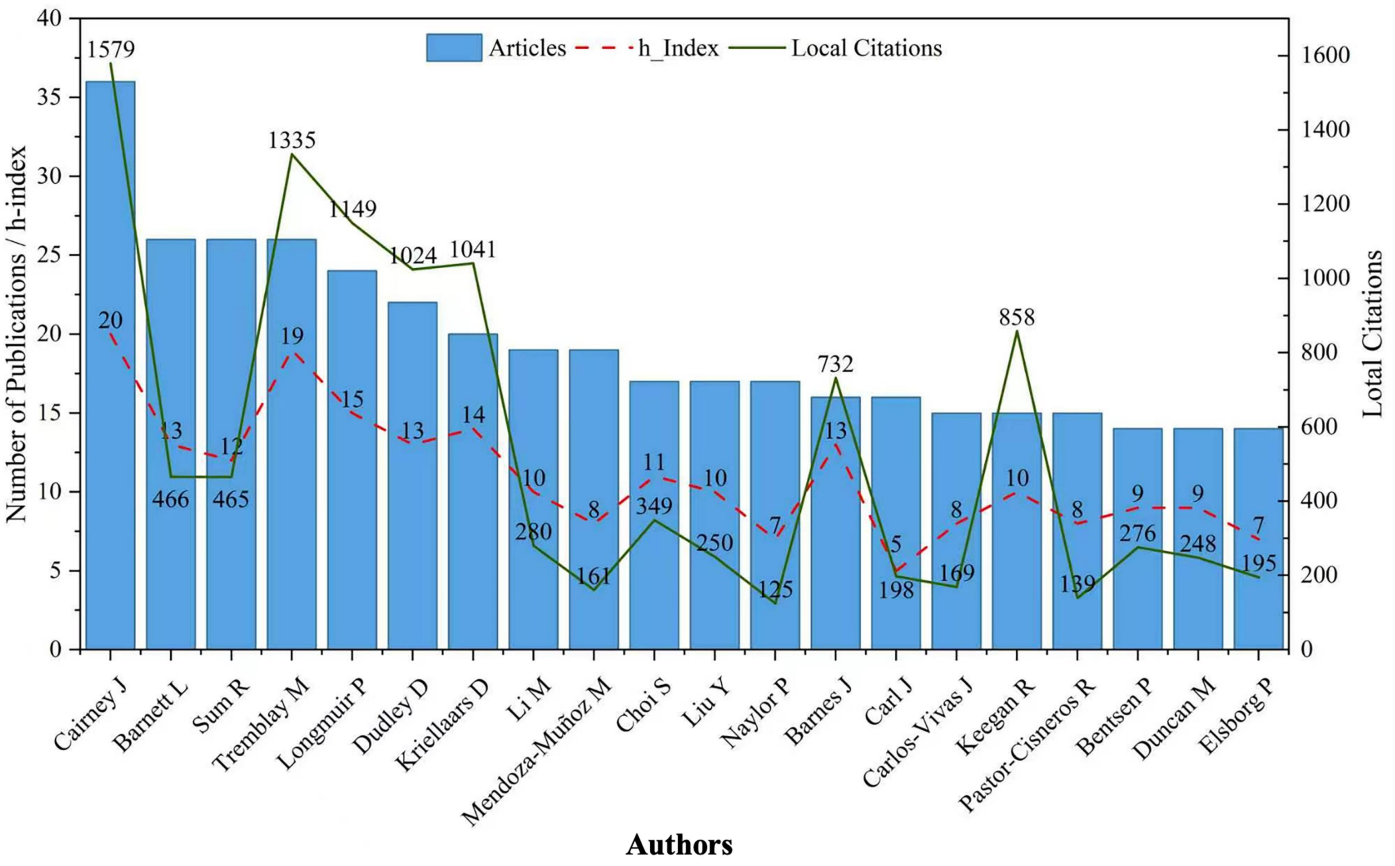
Leading authors in youth physical literacy research: publication output, citation impact, and h-index based on the merged WoSCC–scopus dataset.

The analysis of leading authors revealed notable differences in publication output and academic influence within youth physical literacy research (Figure 6). Based on the merged WoSCC–Scopus dataset, Cairney J ranked first in publication output with 36 articles, followed by Barnett L, Sum R, and Tremblay M (26 articles each), Longmuir *P* (24), Dudley D (22), and Kriellaars D (20). These findings indicate that research productivity in this field is concentrated among a relatively small group of core contributors.

Regarding local citation impact, Cairney J also accumulated the highest number of local citations (Local Citations = 1,579), followed by Tremblay M (1,335), Longmuir *P* (1,149), Kriellaars D (1,041), Dudley D (1,024), Keegan R (858), and Barnes J (732). In terms of h-index, Cairney J ranked first (20), followed by Tremblay M (19), Longmuir *P* (15), Kriellaars D (14), and Barnett L, Dudley D, and Barnes J (13 each).

Several authors demonstrated relatively balanced performance across publication productivity and academic influence. For example, Cairney J combined the highest publication output with the strongest local citation impact and h-index, while Tremblay M and Longmuir *P* also exhibited consistently high performance across multiple indicators. In contrast, authors such as Keegan R and Barnes J accumulated substantial local citation impact despite producing comparatively fewer publications, suggesting that scholarly influence was not always directly proportional to publication output.

Overall, the findings indicate that youth physical literacy research is supported by a relatively small group of highly productive and influential scholars, whose sustained contributions have played important roles in advancing knowledge in the field.

#### 3.7.2 Author collaboration network structure

**Figure 7:**
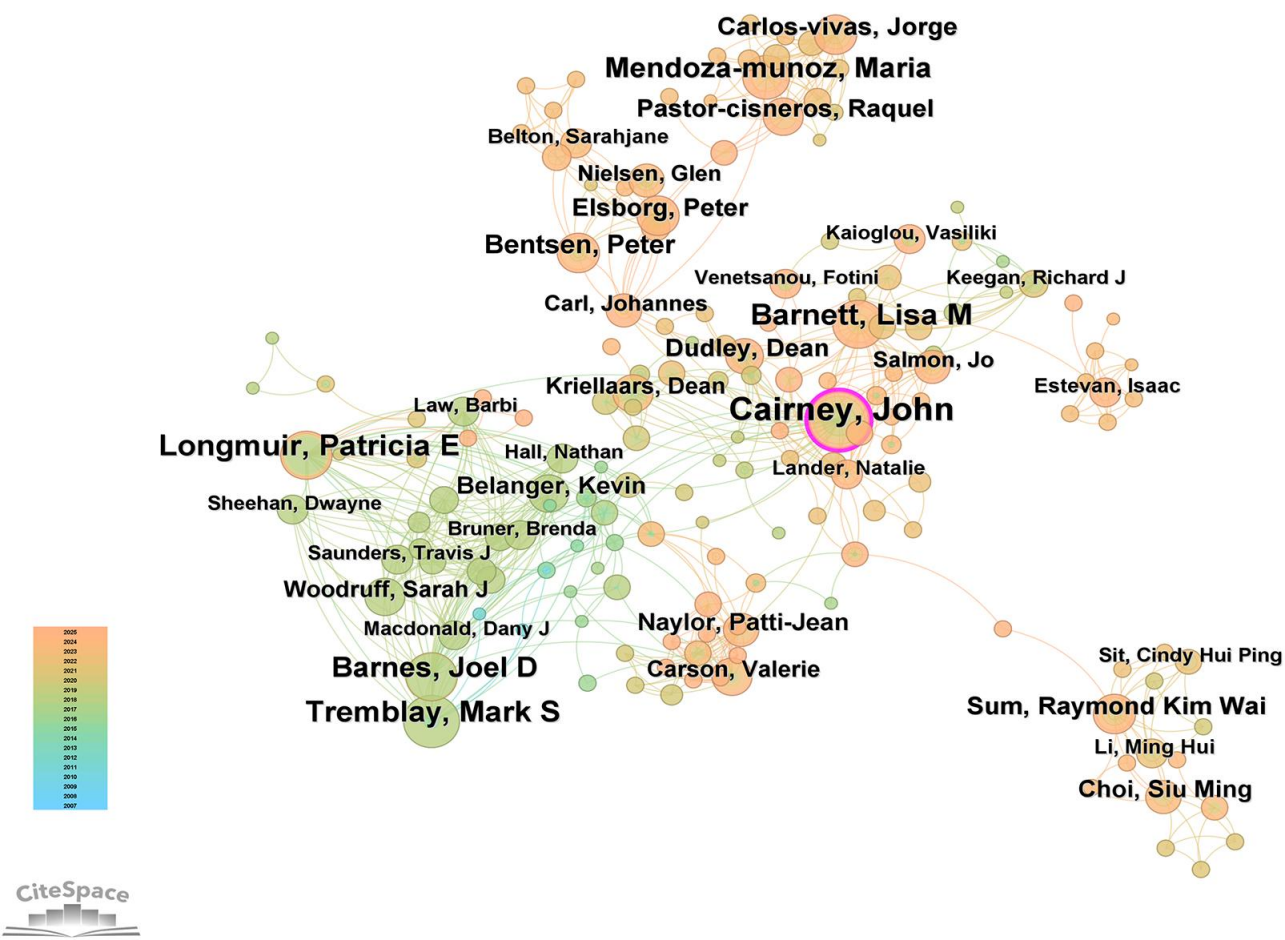
Author collaboration network in youth physical literacy research based on the WoSCC dataset.

**Figure 8:**
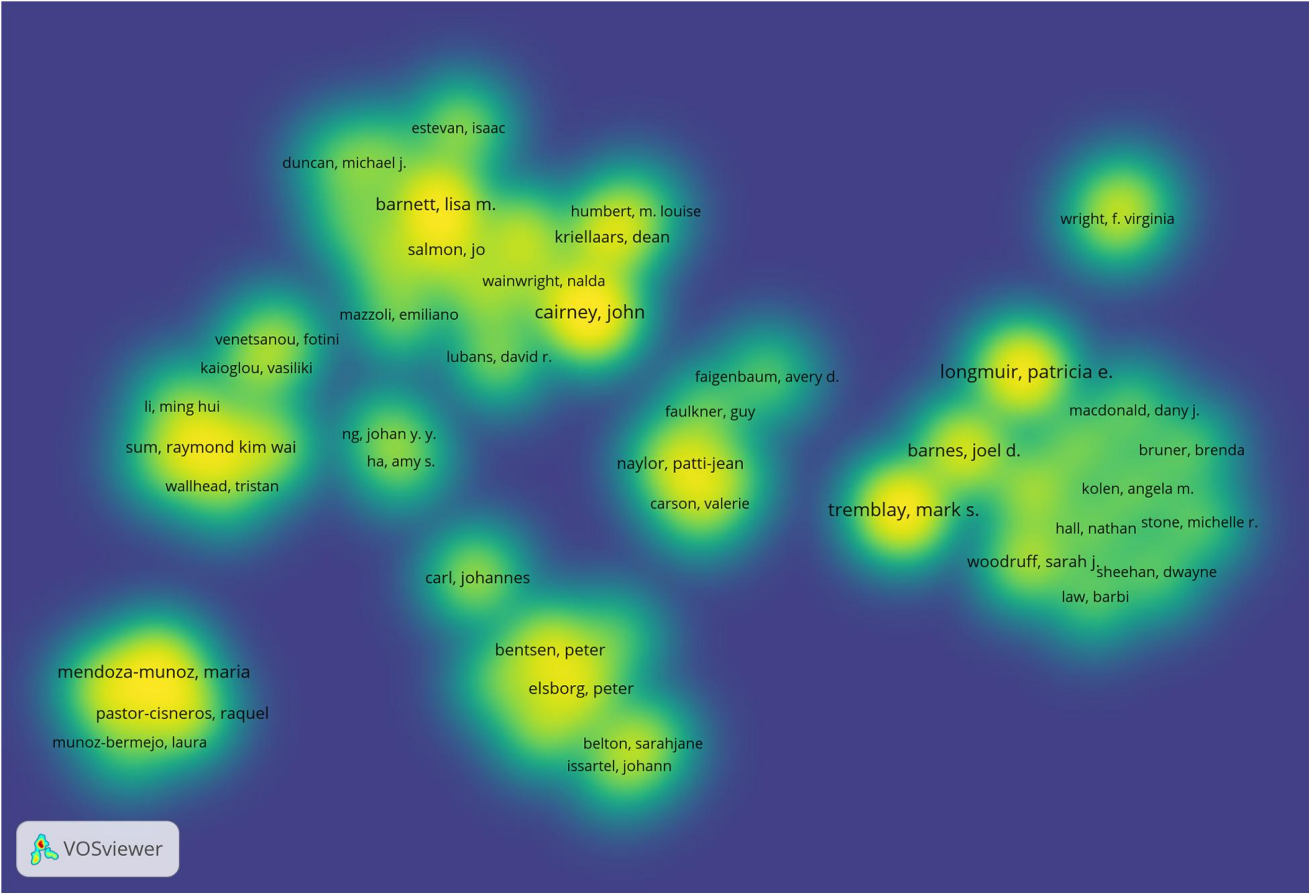
Density visualization of the author collaboration network in youth physical literacy research based on the scopus dataset using VOSviewer.

As shown in Figures 7, 8, the author collaboration network in youth physical literacy research exhibited a decentralized and multi-centered structure, with several visible collaborative groups rather than a single dominant author group.

Figure 7 presents the author collaboration network generated from the WoSCC dataset. Cairney J occupied a prominent position within the network and showed collaborative relationships with Barnett L, Dudley D, Kriellaars D, Salmon J, and other authors. Another visible collaborative group involved Tremblay M, Longmuir P, Barnes J, Belanger K, and Woodruff S. Additional clusters were observed around Sum R, Choi S, and Li M; Mendoza-Muñoz M, Pastor-Cisneros R, and Carlos-Vivas J; and Bentsen P and Elsborg P. These patterns suggest that author collaboration in this field is organized around multiple active research groups.

Figure 8 presents the author density visualization generated from the Scopus dataset using VOSviewer. The highest-density regions were concentrated around authors such as Cairney J, Tremblay M, Longmuir P, Barnett L, and Sum R, indicating that these authors and their collaborators represented major activity areas within the author network. The density visualization generally corresponded to the collaborative clusters observed in Figure 7.

Overall, the combined network and density visualizations indicate that youth physical literacy research is supported by several active author groups and a multi-centered collaboration structure, rather than by a single centralized group.

### 3.8 Keyword co-occurrence and clusters

#### 3.8.1 Keyword co-occurrence analysis

**Figure 9:**
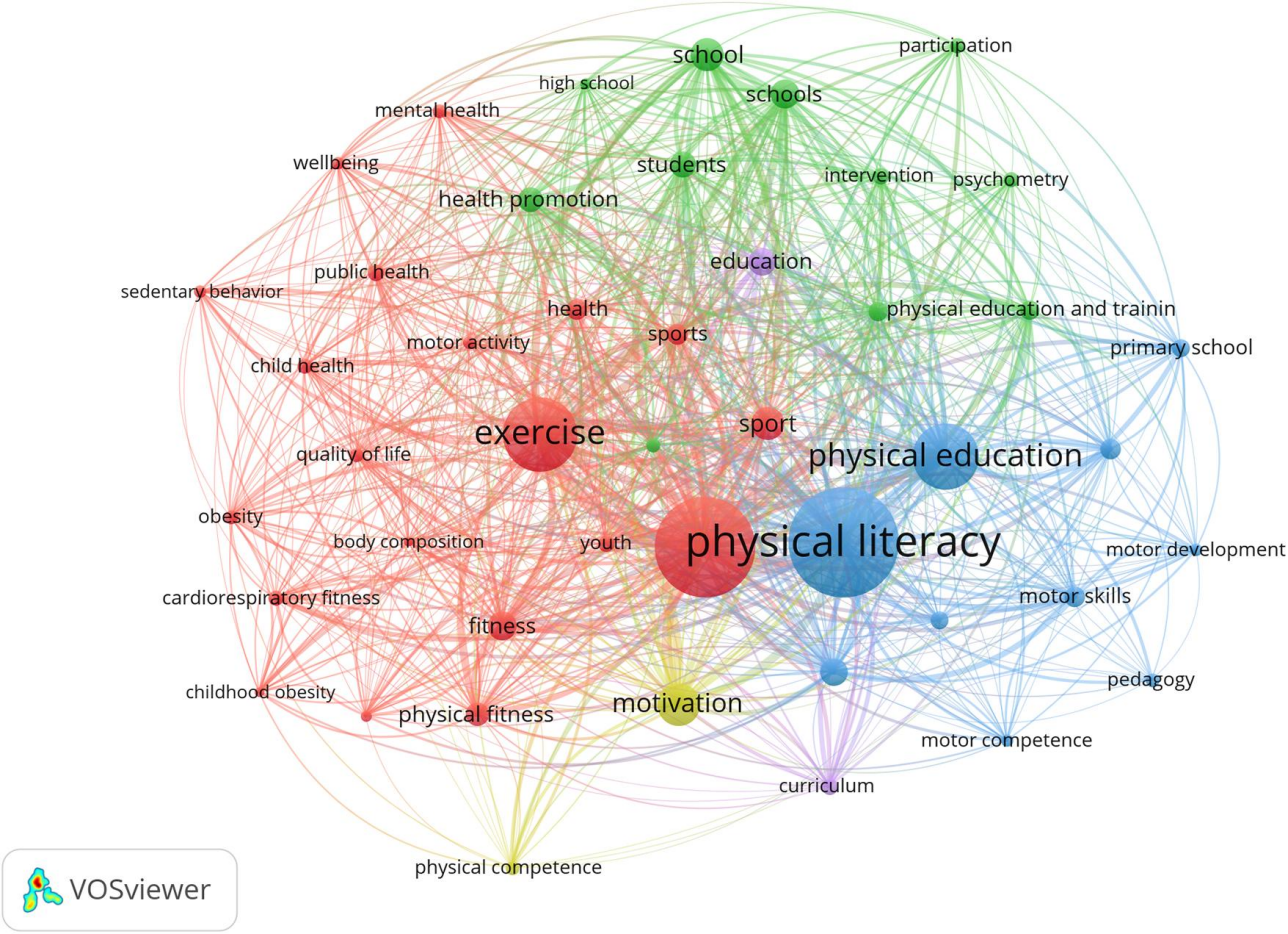
Keyword co-occurrence network in youth physical literacy research based on the scopus dataset using VOSviewer. Different colours represent keyword clusters generated by VOSviewer.

As shown in Figure 9, the keyword co-occurrence network based on the Scopus dataset reveals the major thematic structure of youth physical literacy research. The network is organized into five closely interconnected clusters, indicating substantial conceptual integration across the field.

The most prominent keywords include “physical literacy,” “physical education,” “exercise,” “school,” “motivation,” and “motor competence.” These terms suggest that the field has developed at the intersection of educational practice, physical activity participation, health promotion, and student development.

The blue cluster centers on physical literacy, physical education, motor skills, and motor competence, representing the pedagogical and competence-based foundations of the field. The red cluster emphasizes health-related themes including exercise, fitness, health, obesity, quality of life, mental health, and sedentary behavior, highlighting the growing public health relevance of physical literacy. The green cluster includes school, students, intervention, and participation, reflecting school-based intervention and applied educational contexts. The yellow cluster focuses on motivation and physical competence, representing motivational and competence-related dimensions of physical literacy. The purple cluster contains education and curriculum, reflecting curriculum-related and educational perspectives.

Overall, the findings indicate that youth physical literacy research is organized around five closely connected themes encompassing physical education and motor competence, health and exercise, school-based education, motivation and physical competence, and curriculum-related education. These results further demonstrate the interdisciplinary nature of the field.

#### 3.8.2 Temporal evolution of research topics

**Figure 10:**
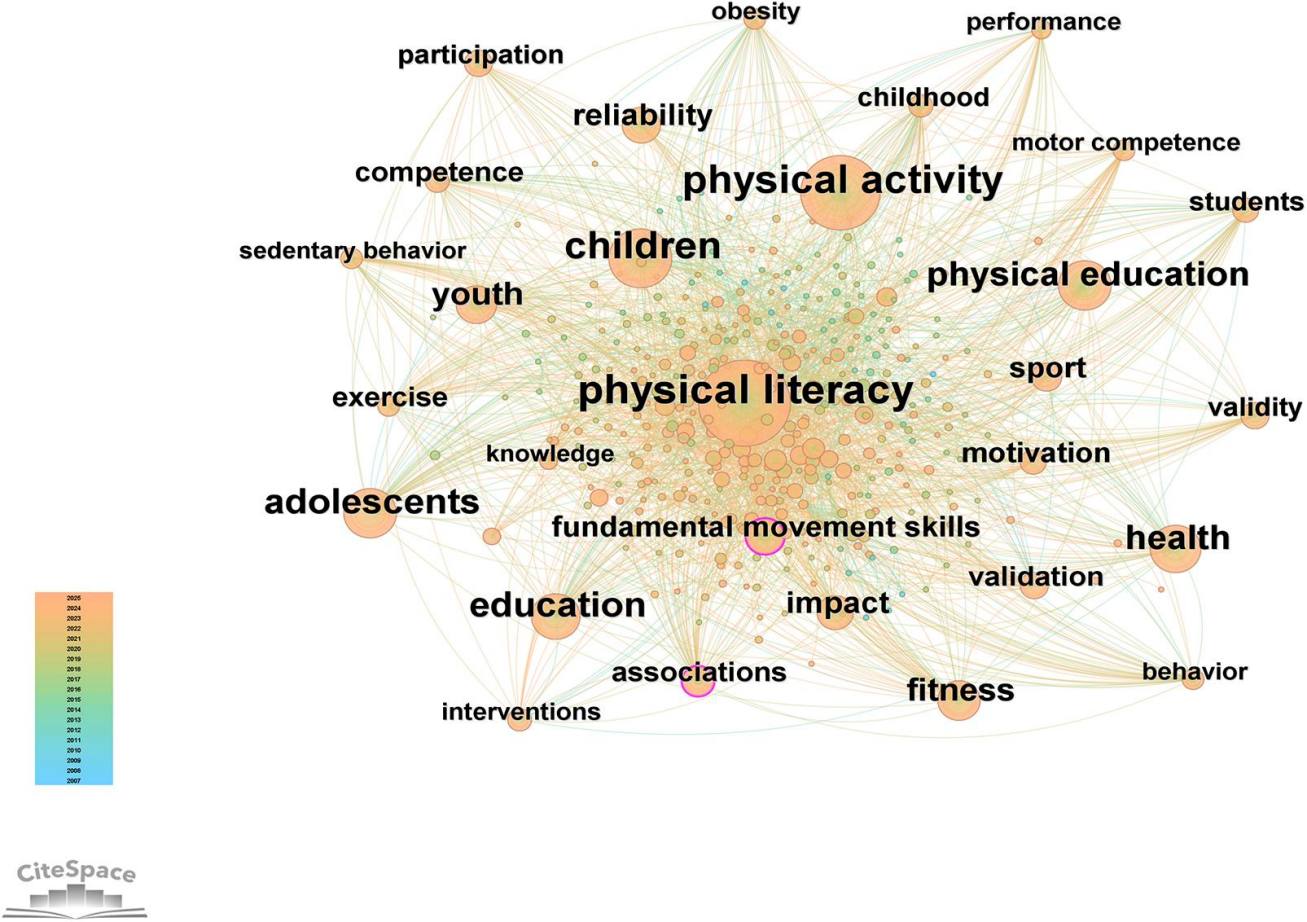
Temporal visualization of keywords showing the evolution of research topics in youth physical literacy research based on the WoSCC dataset using citeSpace. Node colours indicate the temporal distribution of keyword occurrence.

Figure 10 presents the temporal keyword network generated using CiteSpace based on the WoSCC dataset. Node colors represent the average publication year of keywords, allowing the visualization of the temporal evolution of research topics in youth physical literacy.

Earlier research primarily focused on physical education, exercise, childhood, fitness, and physical activity, indicating that the field was initially centered on school-based physical education, physical fitness, and health promotion. During the subsequent development of the field, keywords such as motor competence, physical competence, physical fitness, adolescents, and physical activity became increasingly prominent, reflecting growing attention to motor development, physical competence, and health-related outcomes among young people.

More recent research has expanded toward motivation, validation, reliability, associations, impact, students, and behavior, suggesting increasing interest in psychological mechanisms, measurement and assessment, educational implementation, and evidence-based evaluation. The appearance of these keywords indicates that recent studies have placed greater emphasis on validating measurement instruments and examining the relationships between physical literacy and a wider range of educational and health outcomes.

Overall, the temporal keyword network suggests a progressive evolution from traditional physical education and health-related research toward more comprehensive investigations integrating physical competence, psychological factors, educational practice, and measurement development. This trend reflects the increasing maturity and interdisciplinary development of youth physical literacy research.

#### 3.8.3 Cluster and thematic evolution mapping

**Figure 11:**
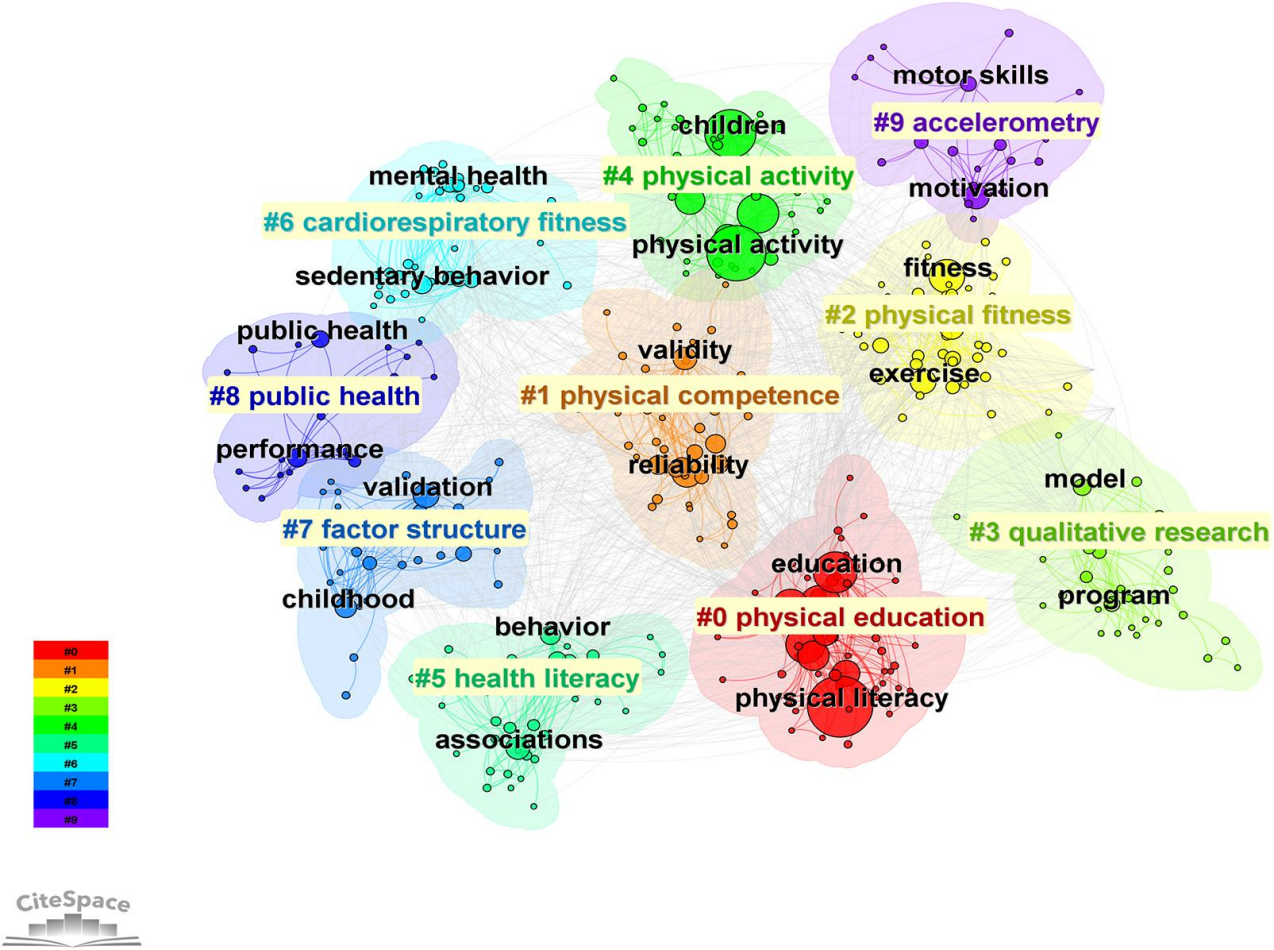
Keyword cluster map showing the thematic structure of youth physical literacy research based on the WoSCC dataset using citeSpace. Cluster numbers indicate CiteSpace-generated keyword clusters.

As shown in Figure 11, the keyword clustering analysis generated using CiteSpace based on the WoSCC dataset reveals the major thematic structure of youth physical literacy research. Compared with the keyword co-occurrence network, the clustering analysis provides a more structured representation of the principal research themes. To facilitate interpretation of the clustering results, the major keyword clusters and their thematic interpretations are summarized in Table 6.

**Table 6:** Major keyword clusters identified in youth physical literacy research and their thematic interpretations.

Cluster ID	Cluster label	Thematic interpretation
#0	Physical education	Educational foundation of youth physical literacy research.
#1	Physical competence	Competence development and measurement of physical literacy.
#2	Physical fitness	Relationships between physical literacy, fitness, and exercise.
#3	Qualitative research	Program implementation and qualitative perspectives.
#4	Physical activity	Participation, active lifestyles, and movement behavior.
#5	Health literacy	Health promotion and health-related behavior.
#6	Cardiorespiratory fitness	Fitness indicators, sedentary behavior, and mental health.
#7	Factor structure	Psychometrics, validation, and assessment tools.
#8	Public health	Population health and public health applications.
#9	Accelerometry	Objective assessment and movement monitoring.

Cluster labels were generated automatically by CiteSpace using the log-likelihood ratio (LLR) algorithm. The overall clustering solution showed Modularity Q=0.4114 and Weighted Mean Silhouette S=0.7478, indicating a reasonably structured and interpretable clustering network. The thematic interpretations were developed by the authors to facilitate understanding of the algorithm-generated cluster labels.

The largest cluster (#0 physical education) represents the core educational context in which physical literacy has been most extensively investigated. Cluster #1 (physical competence) highlights research focusing on competence development, together with related themes such as validity and reliability, reflecting continued interest in assessment and measurement. Cluster #2 (physical fitness) emphasizes exercise- and fitness-related outcomes, whereas Cluster #4 (physical activity) focuses on children and physical activity participation within school and community settings.

Several additional clusters illustrate the expanding scope of the field. Cluster #3 (qualitative research) reflects increasing attention to program implementation and research methodologies. Cluster #5 (health literacy) includes themes such as behavior and associations, indicating growing interest in the relationships between physical literacy and broader health outcomes. Cluster #6 (cardiorespiratory fitness) highlights links between physical literacy and health-related fitness indicators. Cluster #7 (factor structure) primarily represents psychometric research concerning instrument validation and measurement development, while Cluster #8 (public health) demonstrates the integration of physical literacy into broader public health research. Cluster #9 (accelerometry) reflects the application of objective movement-monitoring technologies for assessing physical activity and motor behavior.

Overall, the clustering results indicate that youth physical literacy research has evolved into a multidisciplinary field integrating physical education, physical competence, health promotion, physical activity, measurement and validation, and public health. The coexistence of educational, health, methodological, and technology-oriented themes demonstrates the increasing breadth and maturity of research in this area.

#### 3.8.4 Research frontiers and emerging trends

**Figure 12:**
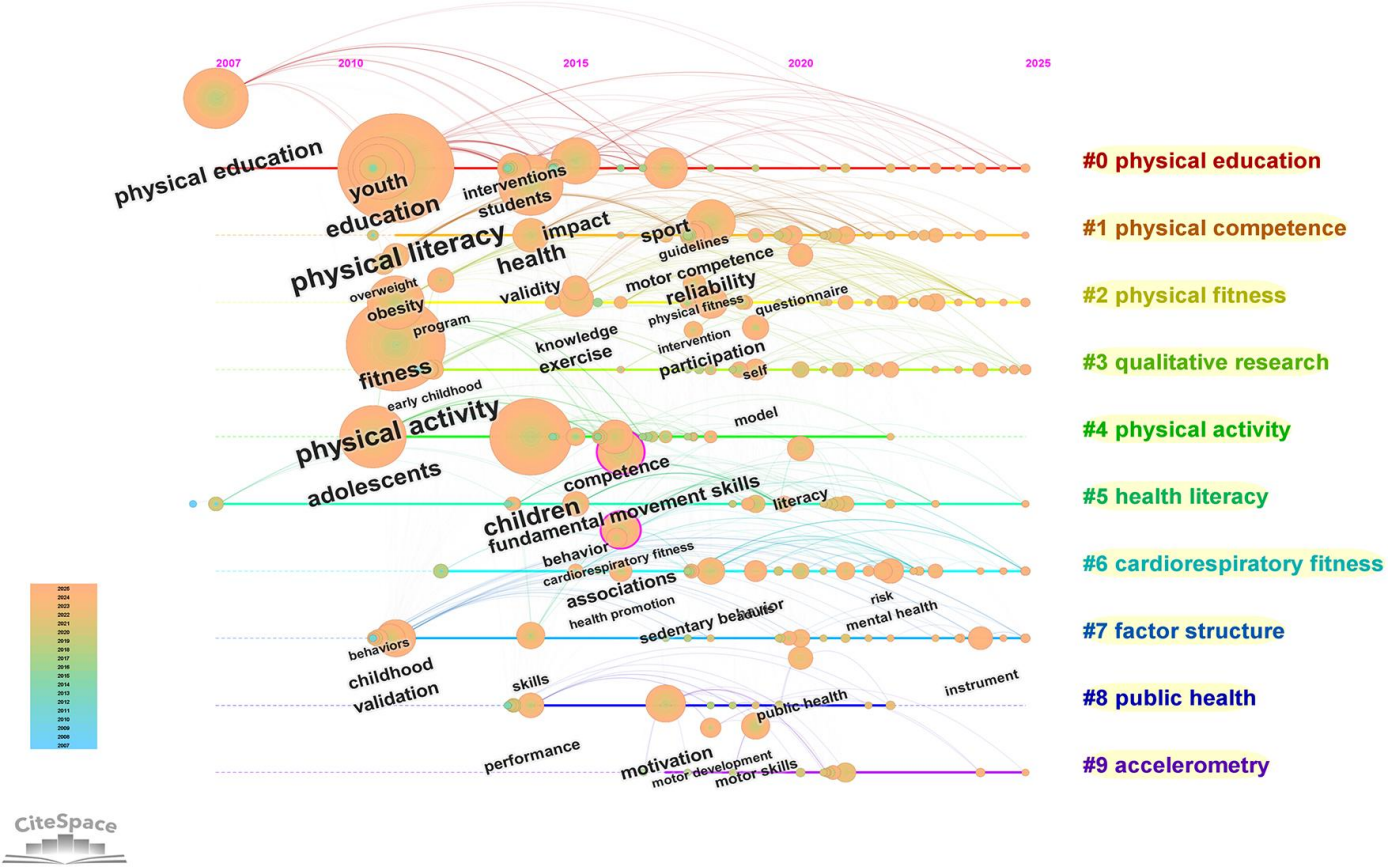
Timeline visualization of keyword clusters in youth physical literacy research based on the WoSCC dataset using citeSpace.

**Figure 13:**
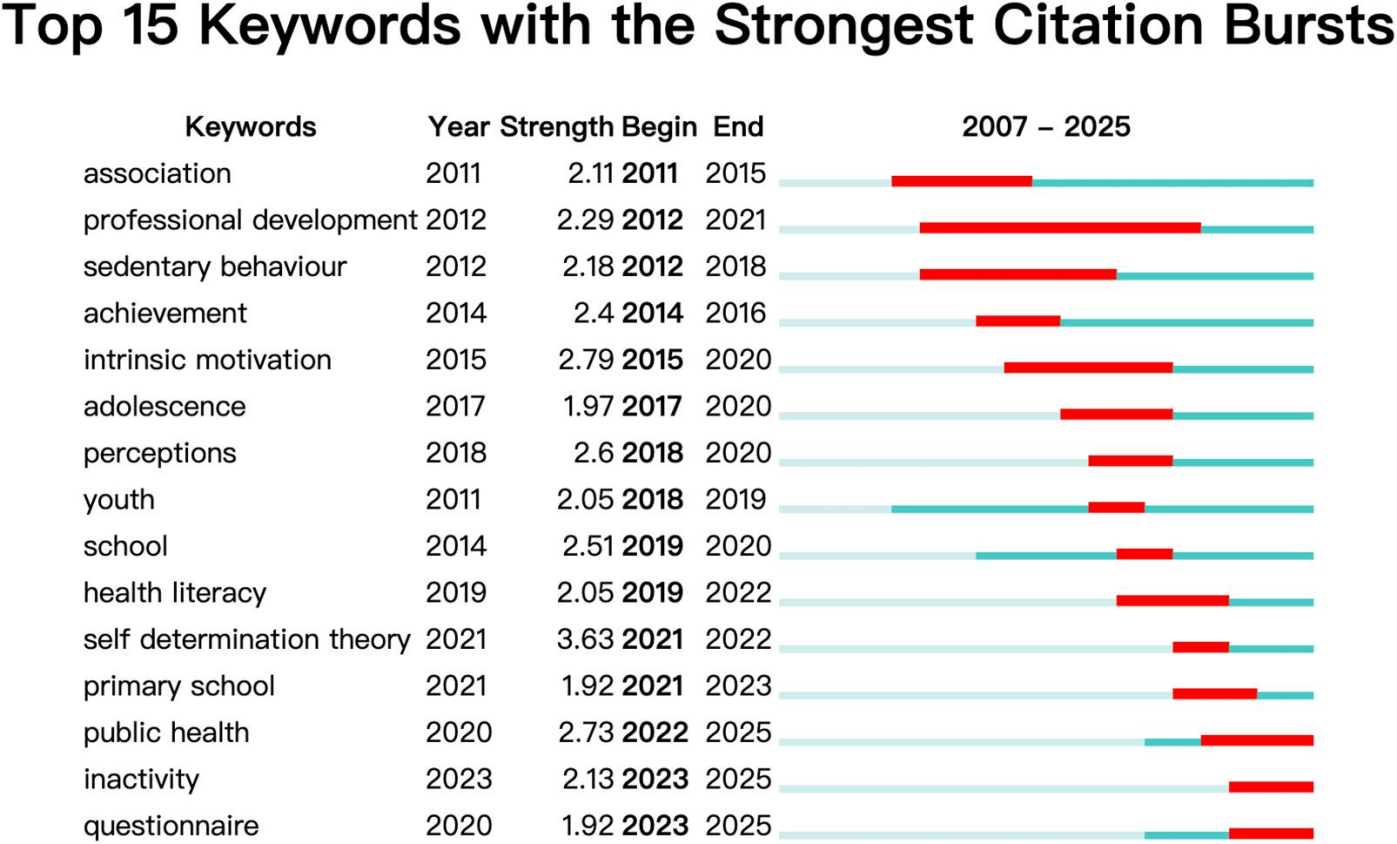
Top 15 keywords with the strongest citation bursts in youth physical literacy research based on the WoSCC dataset using citeSpace.

To illustrate the evolution of research hotspots in youth physical literacy, a keyword timeline map (Figure 12) and a burst keyword map (Figure 13) were generated using CiteSpace.

Figure 12 demonstrates a clear temporal evolution of research themes. Early studies primarily focused on physical education, youth, education, and physical literacy, reflecting the foundational development of the field within educational contexts. As research progressed, increasing attention was directed toward physical activity, children, physical competence, physical fitness, and health-related outcomes, indicating an expansion from educational perspectives to multidimensional investigations of motor development and health. In recent years, topics related to validation, reliability, health literacy, public health, and accelerometry have become increasingly prominent, suggesting growing interest in measurement development, evidence-based assessment, and interdisciplinary applications.

Figure 13 presents the burst keyword analysis, highlighting emerging research frontiers. Early burst keywords, including “association,” “professional development,” “sedentary behaviour,” and “achievement,” primarily reflected foundational research exploring relationships among physical activity, educational practice, and health outcomes. Subsequent bursts, such as “intrinsic motivation,” “adolescence,” “perceptions,” “school,” “health literacy,” and “self-determination theory,” indicate a growing emphasis on psychological mechanisms, educational contexts, and theory-informed research. The most recent burst keywords, including “public health,” “inactivity,” and “questionnaire,” suggest that current research increasingly focuses on population health, sedentary lifestyles, and the development and validation of assessment instruments. To facilitate interpretation of the burst analysis results, the major burst keywords and their temporal characteristics are summarized in Table 7.

**Table 7:** Major burst keywords and their thematic interpretations. .

Keyword	Strength	Burst period	Thematic interpretation
Association	2.11	2011–2015	Early focus on associations between physical literacy and developmental outcomes.
Professional development	2.29	2012–2021	Interest in teacher education, professional learning, and implementation.
Sedentary behaviour	2.18	2012–2018	Concern with inactivity and health-related behavior.
Achievement	2.40	2014–2016	Attention to performance- and achievement-related outcomes.
Intrinsic motivation	2.79	2015–2020	Emphasis on motivational dimensions of physical literacy.
Adolescence	1.97	2017–2020	Growing focus on adolescent populations.
Perceptions	2.60	2018–2020	Attention to psychological and perceptual factors.
Youth	2.05	2018–2019	Consolidation of youth-oriented physical literacy research.
School	2.51	2019–2020	Increased attention to educational and school-based contexts.
Health literacy	2.05	2019–2022	Integration of physical literacy with health promotion perspectives.
Self-determination theory	3.63	2021–2022	Use of motivational and behavioral theoretical frameworks.
Primary school	1.92	2021–2023	Growing attention to school-aged children and primary education.
Public health	2.73	2022–2025	Expansion toward population health and public health applications.
Inactivity	2.13	2023–2025	Recent concern with insufficient physical activity and sedentary lifestyles.
Questionnaire	1.92	2023–2025	Increasing focus on assessment, measurement, and instrument development.

Burst keywords were identified using CiteSpace citation burst detection. Keywords with burst periods extending to 2025, particularly public health, inactivity, and questionnaire, may be considered recent research frontiers in youth physical literacy research.

Taken together, the combined evidence indicates that youth physical literacy research has evolved from early education- and activity-oriented studies toward multidimensional, theory-informed, and measurement-driven research. Current frontiers increasingly emphasize public health applications, health literacy, objective assessment methods, and evidence-based evaluation, reflecting the continued maturation and interdisciplinary expansion of the field.

### 3.9 Three-field plot analysis of authors, institutions, and countries

**Figure 14:**
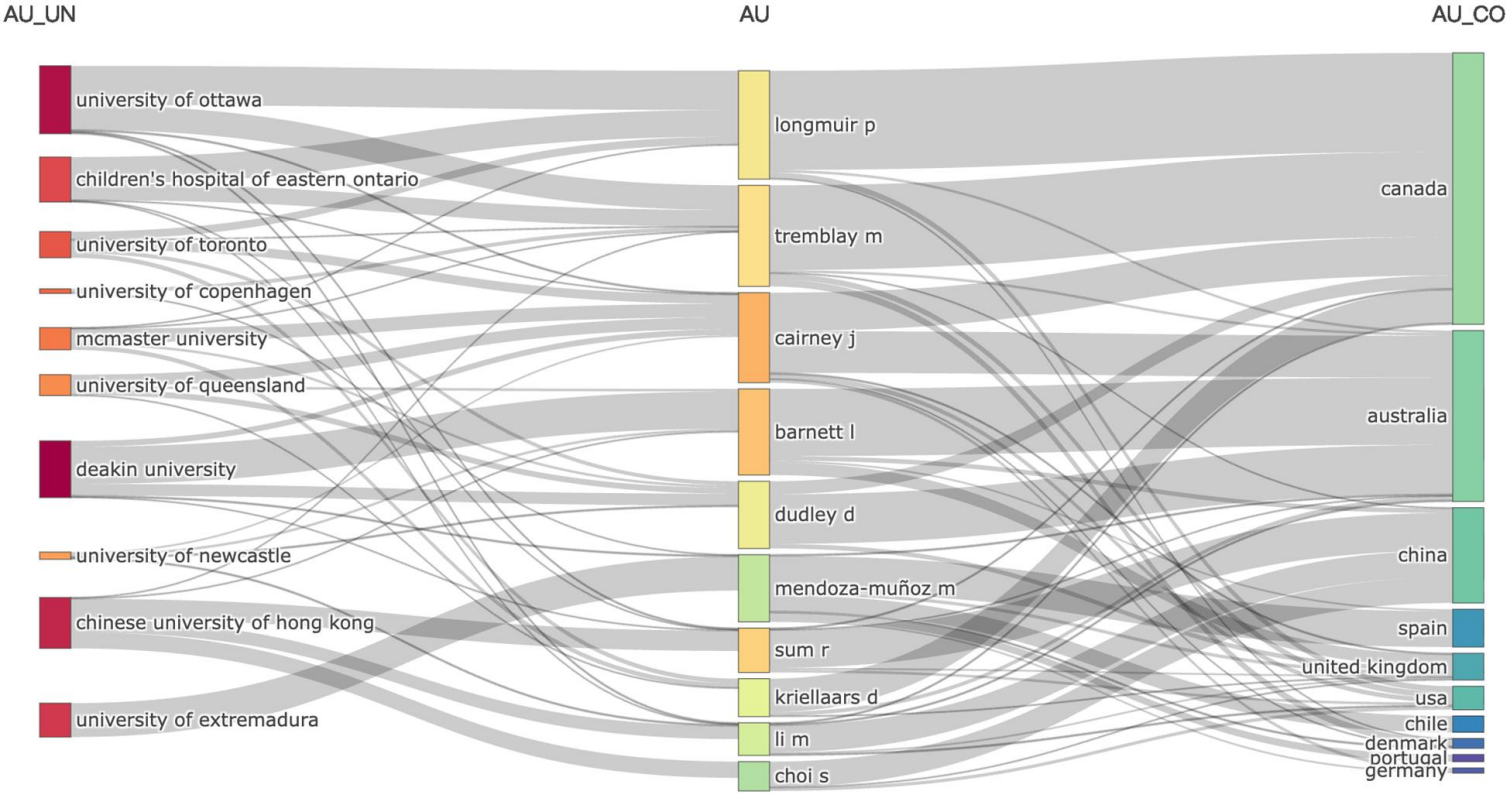
Three-field plot showing relationships among institutions (AU_UN), authors (AU), and countries (AU_CO) in youth physical literacy research based on the merged WoSCC–scopus dataset.

Figure 14 presents a three-field plot illustrating the relationships among institutions (AU_UN), authors (AU), and countries (AU_CO) in youth physical literacy research. Institutions are displayed on the left, authors in the center, and countries on the right, demonstrating the connections between institutional affiliations, leading researchers, and national research contributions.

The plot reveals strong collaborative linkages among leading institutions, prolific authors, and major contributing countries. Canada emerged as the dominant contributor, followed by Australia and China, while additional research contributions were observed from Spain, the United Kingdom, the United States, Chile, Denmark, Portugal, and Germany.

Among the authors, Longmuir P, Tremblay M, Cairney J, Barnett L, and Dudley D were linked with multiple institutions and countries, indicating extensive international collaboration. At the institutional level, the University of Ottawa, Children's Hospital of Eastern Ontario, Deakin University, Chinese University of Hong Kong, University of Toronto, and University of Extremadura were prominently linked with highly productive authors and major contributing countries, highlighting their central positions within the global research network.

Overall, the three-field plot suggests a geographically concentrated yet internationally connected research landscape, with a relatively small number of leading institutions and researchers contributing substantially to the global development of youth physical literacy research through extensive cross-national collaboration.

## 4 Discussion

### 4.1 General research profile

This study provides a comprehensive bibliometric overview of youth physical literacy research from 2000 to 2025. The findings reveal a field that has expanded rapidly in both publication volume and conceptual scope. The strong consistency observed between the WoSCC and Scopus datasets further supports the robustness of the major publication trends and thematic patterns identified in this study.

The annual growth in publications indicates increasing international attention to physical literacy as a multidimensional construct relevant not only to movement competence, but also to broader developmental, educational, psychological, and health-related outcomes. In this sense, the field has evolved from a relatively specialized topic into a more visible interdisciplinary area of inquiry.

As shown in Figure 2, publication output remained limited before 2010, increased gradually between 2010 and 2015, and then accelerated markedly after 2015. This trajectory likely reflects the consolidation of physical literacy as a broader conceptual framework encompassing competence, motivation, confidence, and sustained engagement in physical activity (13, 22). The development of more standardized assessment frameworks during this period may also have supported the expansion of empirical research by improving the operationalization and measurement of the construct (13). This interpretation is further supported by the increasing prominence of validation-, reliability-, and assessment-related themes identified in the keyword analyses.

This growth also appears to be associated with increasing global concern over insufficient physical activity and sedentary behavior among children and adolescents (23–25). Within this context, physical literacy has been increasingly positioned as a framework linking early movement experiences with long-term health, behavioral, and developmental outcomes (26). The growing prominence of themes such as obesity, sedentary behavior, health, and public health further supports this interpretation, suggesting that the field has become increasingly aligned with public health priorities (27–29). At the same time, this expansion has clear relevance for educational agendas, as schools remain one of the primary settings in which physical literacy can be developed and linked to broader developmental and well-being-related outcomes.

At the country level, the research landscape is dominated by a relatively small number of contributors, particularly Canada, Australia, and China, which occupy leading positions in both publication output and international collaboration networks (Table 4, Figure 3). This pattern may partly reflect differences in national policy support and institutional investment in physical education, physical activity promotion, and public health. For example, the Australian Physical Literacy Framework emphasized a shared national language for physical literacy development across sectors (30), while Canadian initiatives have promoted integrated movement behaviors and lifelong participation through coordinated national strategies (31). In China, policies such as *Healthy China 2030* and the National Fitness Program have further strengthened the link between physical activity promotion and public health goals (32). These policy contexts may partly explain the prominent roles of these countries in shaping the development of the field.

At the institutional level, leading contributions are concentrated in universities and research centers specializing in physical education, kinesiology, and health sciences (Figure 4, Figure 5). The dense collaboration networks observed among these institutions suggest the presence of stable research groups and sustained interdisciplinary and international cooperation. Such patterns are consistent with previous bibliometric evidence showing that established institutions often function as hubs for knowledge production and collaboration (33, 34). A similar concentration is also evident at the author level, where a relatively small core group of influential scholars, including Cairney J, Tremblay M, Longmuir P, and Barnett L, contribute substantially to both research productivity and international collaboration (Figures 6–8). This concentration is typical of a field moving from emergence toward greater consolidation, in which key scholars and institutions play central roles in defining research directions.

Overall, the general research profile suggests that youth physical literacy research is becoming increasingly global, collaborative, and interdisciplinary. Importantly, the field appears to be shifting beyond a narrow focus on physical competence toward broader health, educational, and psychosocial concerns. This evolution is particularly relevant to current efforts to promote positive developmental and health-related outcomes through school-based physical activity, as reflected by the increasing prominence of school-, health-, and motivation-related themes identified in the bibliometric analyses. Physical literacy may therefore be positioned not merely as a concept within physical education, but increasingly as an interdisciplinary framework for promoting healthy youth development across educational and public health contexts.

### 4.2 Research hotspots and trends

#### 4.2.1 Research hotspots: keyword co-occurrence and clustering

As shown in Figure 9, the keyword co-occurrence network reveals the core thematic structure of youth physical literacy research. High-frequency keywords such as physical literacy, physical education, exercise, school, motivation, and motor competence occupy central positions, indicating that the field is strongly oriented toward the relationships among physical competence, educational practice, health promotion, and physical activity behaviors. This pattern is consistent with previous research emphasizing that physical literacy is closely linked to physical activity behaviors and health outcomes (35, 36). For example, Caldwell et al. highlighted that motor competence and physical literacy are key determinants of children's physical activity participation and health development (35), while Carl et al. further demonstrated that physical literacy is associated with obesity-related indicators and overall health status among children and adolescents (36). The dense interconnections among these keywords further suggest that youth physical literacy research has developed into a highly integrated and multidisciplinary domain, linking physical competence with broader educational, behavioral, and well-being-related agendas.

Building upon this, the keyword clustering analysis (Figure 11) provides a more structured understanding of thematic distributions. The largest clusters include #0 physical education, #1 physical competence, #2 physical fitness, and #4 physical activity, which together form the conceptual foundation of the field. These clusters indicate that physical literacy research is grounded in educational contexts while increasingly integrating physical competence, physical activity, and health-related outcomes. This finding aligns with existing literature suggesting that school-based physical education serves as a primary context for developing motor competence and physical literacy (37, 38). At the same time, the prominence of these education-related clusters also suggests that schools remain a central setting in which physical literacy may be linked to broader student development and related well-being outcomes.

Several additional clusters further demonstrate the expanding scope of the field. For example, Cluster #5 (health literacy) reflects increasing attention to the relationships between physical literacy and broader health outcomes, supporting the growing recognition that physical literacy should be considered within broader health promotion frameworks rather than solely within physical education (39–41). Cluster #6 (cardiorespiratory fitness) further highlights the close association between physical literacy and health-related fitness, while Cluster #7 (factor structure) indicates increasing emphasis on the development and psychometric validation of assessment instruments. Furthermore, Cluster #8 (public health) and Cluster #9 (accelerometry) suggest growing interest in population health applications and the use of objective technologies to assess physical activity and movement behavior. Together, these findings indicate that youth physical literacy research is expanding beyond traditional educational contexts toward more comprehensive approaches integrating health promotion, measurement, and broader developmental outcomes. This trend is also consistent with growing evidence suggesting that physical activity, together with physical literacy-related factors, is associated with motivation, cognitive function, academic performance, and broader developmental outcomes among children and adolescents (42).

Taken together, these thematic patterns suggest that youth physical literacy research has evolved from a predominantly education-based framework toward a broader interdisciplinary field integrating physical education, health promotion, measurement science, and public health. This evolution further supports the role of physical literacy as a comprehensive framework for promoting healthy youth development through school-based physical activity.

#### 4.2.2 Research evolution: timeline analysis

Figure 12 illustrates the temporal evolution of research themes, providing a longitudinal view of how key topics in youth physical literacy have developed over time. In the early stage (pre-2010), research was primarily centered on physical education, youth, education, and physical literacy, reflecting an initial focus on foundational concepts and educational contexts.

Between 2010 and 2015, research attention gradually shifted toward physical activity, children, physical competence, and physical fitness. This transition reflects the increasing operationalization of physical literacy through measurable components and the growing emphasis on promoting physical activity and competence development among children and adolescents (13, 38, 43).

Following 2015, the field entered a phase of rapid expansion and thematic diversification. Topics such as validation, reliability, health literacy, and public health became increasingly prominent, indicating a movement from descriptive research toward more rigorous measurement, interdisciplinary applications, and broader health promotion perspectives. This development aligns with growing recognition of the importance of addressing behavioral and psychosocial determinants of physical activity in youth populations (36, 44, 45).

In more recent years, increasing attention has also been directed toward validation, reliability, and objective assessment approaches, reflecting continued efforts to improve the measurement of physical literacy across different populations (46). At the same time, physical literacy is increasingly being conceptualized as a multidimensional construct with implications extending beyond physical competence to broader health and developmental outcomes, including mental health and psychosocial outcomes (11, 47). Such developments suggest that researchers are increasingly interested in understanding how physical literacy contributes to healthy development within educational and broader public health contexts. Overall, the timeline analysis indicates a progression from foundational and measurement-oriented research toward increasingly interdisciplinary, school-relevant, and health-oriented approaches.

#### 4.2.3 Emerging trends: burst keyword analysis

To further identify emerging research frontiers, Figure 13 presents the burst keyword analysis, revealing periods of intensified scholarly attention to specific topics. Early burst terms such as association, professional development, sedentary behaviour, and achievement reflect an initial focus on the relationships between physical literacy, educational practice, health-related behaviors, and performance-related outcomes.

Subsequently, keywords including intrinsic motivation, adolescence, perceptions, school, health literacy, and self-determination theory exhibited citation bursts, indicating increasing attention to the psychological, developmental, and contextual dimensions of physical literacy research. This shift suggests growing recognition that motivation, individual perceptions, adolescent development, and school-related environments play important roles in shaping physical activity behaviors and long-term engagement (48, 49).

More recently, burst terms such as primary school, public health, inactivity, and questionnaire have emerged, indicating increasing attention to school-based contexts, population health, sedentary lifestyles, and the development of more refined assessment tools. In particular, the prominence of questionnaire reflects ongoing efforts to improve measurement precision, which is essential for advancing empirical research and strengthening the reliability and validity of physical literacy assessment (50–52).

Taken together, the burst analysis indicates a movement toward a more comprehensive framework that integrates physical, psychological, behavioral, educational, and public health dimensions. Importantly, the emergence of terms such as school, primary school, public health, inactivity, and questionnaire suggests that youth physical literacy research is becoming increasingly relevant to school-based and health-oriented efforts to promote broader developmental and well-being-related outcomes through physical activity, as reflected by the recent emergence of school-, public health-, and assessment-related burst keywords, extending beyond traditional physical education outcomes.

### 4.3 Educational and developmental implications of youth physical literacy research

Compared with previous bibliometric reviews that primarily mapped the broader physical literacy literature (15, 16), the present analysis more clearly identifies youth-specific thematic developments. The keyword clustering, timeline, and burst analyses indicate a shift from earlier emphases on physical education, motor competence, and physical fitness toward motivation, self-determination theory, health literacy, public health, questionnaire development, and assessment validation. Recent burst keywords such as public health, inactivity, and questionnaire further suggest that youth physical literacy research is increasingly connected with population health, sedentary behaviour, and measurement development. These findings extend previous bibliometric reviews by demonstrating that recent youth physical literacy research has increasingly incorporated motivational, health-literacy, and assessment-related perspectives that were less prominent in earlier reviews of the broader physical literacy literature.

The findings of this study have important implications for advancing school-based physical activity beyond traditional physical education models. First, the prominence of keywords such as physical education, physical competence, physical activity, motivation, health literacy, and school suggests that physical literacy research is increasingly aligned with multidimensional approaches to student development. This indicates that school-based physical activity initiatives should not be designed solely to improve physical fitness or sport skills, but should also address psychological, behavioral, educational, and health-related dimensions of student well-being, consistent with multidimensional definitions of physical literacy and recent evidence linking physical literacy with broader youth development outcomes (13, 22, 35, 36).

Second, the emergence of validation, reliability, health literacy, self-determination theory, questionnaire, and public health as recent research frontiers highlights the need for more theory-informed and evidence-based school interventions. Future school-based physical activity programs may benefit from integrating physical competence development with motivational support, health literacy, and lifelong physical activity engagement while adopting valid and reliable assessment approaches. Such strategies are consistent with self-determination theory and growing evidence supporting the roles of physical literacy in promoting healthy youth development (48, 49, 51, 53).

Third, the increasing attention to measurement, validation, and objective assessment suggests that schools and researchers require reliable, age-appropriate, and context-sensitive tools to evaluate physical literacy and related well-being outcomes. Such tools are essential for assessing whether school-based physical activity programs contribute not only to physical competence, but also to broader student well-being, particularly given ongoing concerns regarding the validity, reliability, and feasibility of physical literacy assessments in school-aged populations (22, 46, 50, 54).

From a broader policy perspective, these findings may also be interpreted through selected Sustainable Development Goals (SDGs). The increasing emphasis on health promotion, physical activity, and well-being aligns with SDG 3 (Good Health and Well-Being), while the prominence of school-based physical literacy and educational contexts relates to SDG 4 (Quality Education). In addition, the concentration of publications in high-income and predominantly English-speaking countries suggests that global knowledge production in physical literacy remains unevenly distributed, which may limit the representation of diverse cultural, linguistic, and educational contexts and highlights the importance of broader international participation consistent with SDG 10 (Reduced Inequalities) (15, 16). Therefore, although the present study provides a broad overview of global research trends, the findings should be interpreted with caution because the literature remains heavily concentrated in English-language and high-income-country contexts, which may underrepresent perspectives from low- and middle-income regions.

Collectively, these findings support a shift from traditional physical education models toward more integrated, well-being-oriented school physical activity frameworks. Physical literacy may serve as a useful conceptual framework for integrating movement competence, motivation, health literacy, and lifelong engagement in physical activity, while supporting broader educational and public health objectives.

### 4.4 Strengths, limitations, and future directions

This study has several notable strengths. First, it provides a comprehensive and systematic bibliometric analysis of youth physical literacy research based on a mixed-source dataset integrating the Web of Science Core Collection and Scopus, covering the period from 2000 to 2025. By integrating multiple bibliometric tools, the study offers a multidimensional perspective on publication trends, collaboration networks, and research hotspots. The combined use of co-occurrence analysis, clustering, timeline visualization, and burst detection further strengthens the findings and enables a more nuanced understanding of the intellectual structure and thematic evolution of the field. The strong consistency observed between the WoSCC and Scopus datasets further enhances the robustness of the identified publication trends, collaboration patterns, and thematic evolution.

Despite these strengths, several limitations should be acknowledged. First, although this study integrated WoSCC and Scopus, relevant publications indexed exclusively in other databases such as PubMed, ERIC, or Google Scholar may still have been excluded. Because duplicate records from WoSCC and Scopus were consolidated and affiliation metadata were standardized during data cleaning, country-level publication frequencies in the merged dataset may differ slightly from those reported in individual databases. However, this limitation is unlikely to affect the overall ranking patterns or substantive conclusions of the study. Second, the analysis was limited to English-language publications, which may have introduced language bias and underrepresented research from non-English-speaking regions. In addition, because student well-being and related concepts were not included as direct search terms, conclusions regarding these themes should be interpreted as emerging patterns identified through bibliometric analyses rather than predefined search targets. Third, as with all bibliometric studies, the findings depend on the quality and completeness of database records, and citation-based indicators may not fully capture the practical or educational impact of physical literacy research. Finally, the interpretation of clustering patterns and keyword evolution inevitably involves a degree of subjectivity, which may have influenced the categorization of research themes.

Based on these limitations, several directions for future research can be proposed. First, future bibliometric studies could incorporate multiple databases and multilingual sources to provide a more comprehensive representation of global research (17). Second, there is a need for more longitudinal and intervention-based empirical studies to examine how physical literacy relates to student well-being outcomes, particularly within school-based physical activity contexts and across diverse cultural and educational settings (53, 55). Third, further efforts should be made to develop and validate standardized, context-sensitive assessment tools that can improve the comparability, reliability, and practical applicability of physical literacy measurement in educational settings (54). Finally, future research may benefit from greater interdisciplinary integration, particularly across public health, education, psychology, and sport science, to better understand how the physical, psychological, social, and educational dimensions of physical literacy interact to support student development and well-being beyond traditional physical education models (56).

## 5 Conclusions

This study provides a comprehensive bibliometric overview of youth physical literacy research from 2000 to 2025, based on an integrated analysis of records retrieved from the Web of Science Core Collection and Scopus databases. The findings show that youth physical literacy research has expanded rapidly, particularly after 2015, and has developed into an increasingly interdisciplinary field connecting physical education, physical competence, health promotion, health literacy, motivation, public health, and school-based physical activity.

The thematic evolution of the field suggests a shift from traditional emphases on physical competence and motor skill development toward broader educational, behavioral, health, and well-being-oriented perspectives. Recent research frontiers, including health literacy, public health, self-determination theory, questionnaire development, and measurement, assessment, and validation, suggest that youth physical literacy is increasingly relevant to school-based strategies for promoting holistic youth development.

Overall, the present study highlights physical literacy as a useful framework that may inform future research, educational practice, and policy discussions related to school-based physical activity. Future research should prioritize longitudinal and intervention-based designs, valid, reliable, and context-sensitive assessment tools, and interdisciplinary approaches to better understand how physical literacy can support the physical, psychological, social, and educational dimensions of youth development.

## Data Availability

The raw data supporting the conclusions of this article will be made available by the authors, without undue reservation.

## References

[1] Carcelén-Fraile MD Aibar-Almazán A Hita-Contreras F. Aerobic training on mental health in children and adolescents: a systematic review with meta-analysis. Appl Sci. (2025) 15:9572. 10.3390/app15179572

[2] Berki T Csányi T Tóth L. Associations of physical activity and physical education enjoyment with self-concept domains among Hungarian adolescents. BMC Psychol. (2024) 12(1):449 10.1186/s40359-024-01953-w 39169414 PMC11340068

[3] Hawani A Chikha A Souissi M Trabelsi O Mrayah M Souissi N. The feeling of pleasure for overweight children during different types of physical activity. Children. (2023) 10(9):1526. 10.3390/children10091526 37761487 PMC10528862

[4] Zamorano-García D Infantes-Paniagua Á Cuevas-Campos R Fernández-Bustos JG. Impact of physical activity-based interventions on children and Adolescents' Physical self-concept: a meta-analysis. Res Q Exerc Sport. (2023) 94(1):1–14. 10.1080/02701367.2021.1927945 34860643

[5] Gmmash A Alonazi A Almaddah M Alkhateeb A Sabir O Alqabbani S. Influence of an 8-week exercise program on physical, emotional, and mental health in Saudi adolescents: a pilot study. Medicina (B Aires). (2023) 59(5):883. 10.3390/medicina59050883 PMC10223168 37241115

[6] Recchia F Bernal JDK Fong DY Wong SHS Chung PK Chan DKC. Physical activity interventions to alleviate depressive symptoms in children and adolescents. JAMA Pediatr. (2023) 177(2):132. 10.1001/jamapediatrics.2022.5090 36595284 PMC9857695

[7] Monteiro D Rodrigues F Lopes V. Social support provided by the best friend and vigorous-intensity physical activity in the relationship between perceived benefits and global self-worth of adolescents. Revista de Psicodidáctica (English ed.). (2021) 26(1):70–7. 10.1016/j.psicoe.2020.11.004

[8] Vaquero-Solís M Tapia-Serrano MA Hortigüela-Alcalá D Sierra-Díaz MJ Sánchez-Miguel PA. Physical activity and quality of life in high school students: proposals for improving the self-concept in physical education. Int J Environ Res Public Health. (2021) 18(13):7185. 10.3390/ijerph18137185 34281121 PMC8297227

[9] Till K Lara-Bercial S Baker J Morley D. Challenges and solutions to supporting physical literacy within youth sport. Sports Med. (2025) 55(12):2953–65. 10.1007/s40279-025-02313-3 40960718 PMC12628402

[10] Çinar M Hassani F. The effects of traditional games on physical literacy among school-aged children. Front Sports Act Living. (2026) 7:1759890. 10.3389/fspor.2025.1759890 41624346 PMC12855449

[11] Shanshan Z Ping T Jiabin L Tianzhuo L Xiaomei L Bolei W. Relationship between physical literacy and mental health in adolescents: a moderated mediation model with resilience and physical activity as variables. Front Psychol. (2025) 16:1518423. 10.3389/fpsyg.2025.1518423 40008347 PMC11854620

[12] Liu X Wang D. Examining the interplay of physical literacy, learner engagement, and physical education satisfaction in fostering overall well-being. Acta Psychol (Amst). (2026) 265:106582. 10.1016/j.actpsy.2026.106582 41863884

[13] Shearer C Goss HR Edwards LC Keegan RJ Knowles ZR Boddy LM. How is physical literacy defined? A contemporary update. J Teach Phys Educ. (2018) 37(3):237–45. 10.1123/jtpe.2018-0136

[14] Bailey R Glibo I Koenen K Samsudin N. What is physical literacy? An international review and analysis of definitions. Kinesiology Review. (2023) 12(3):247–60. 10.1123/kr.2023-0003

[15] Urbano-Mairena J Castillo-Paredes A Muñoz-Bermejo L Denche-Zamorano Á Rojo-Ramos J Pastor-Cisneros R. A bibliometric analysis of physical literacy studies in relation to health of children and adolescents. Children. (2023) 10(4):660. 10.3390/children10040660 37189909 PMC10137032

[16] Qian H Ren X Wang H Zou Y. Global insights and key trends in physical literacy research: a bibliometric analysis and literature review from 2007 to 2024. J Multidiscip Healthc. (2025) 18:2039–55. 10.2147/JMDH.S515715 40242081 PMC12002333

[17] Aria M Cuccurullo C. Bibliometrix: an R-tool for comprehensive science mapping analysis. J Informetr. (2017) 11(4):959–75. 10.1016/j.joi.2017.08.007

[18] Lim WM Kumar S Donthu N. How to combine and clean bibliometric data and use bibliometric tools synergistically: guidelines using metaverse research. J Bus Res. (2024) 182:114760. 10.1016/j.jbusres.2024.114760

[19] Shen X Wang L. Topic evolution and emerging topic analysis based on open source software. J Data Inf Sci. (2020) 5(4):126–36. 10.2478/jdis-2020-0033

[20] Liu Z Yin Y Liu W Dunford M. Visualizing the intellectual structure and evolution of innovation systems research: a bibliometric analysis. Scientometrics. (2015) 103(3):799–820. 10.1007/s11192-014-1517-y

[21] Pan X Yan E Cui M Hua W. Examining the usage, citation, and diffusion patterns of bibliometric mapping software: a comparative study of three tools. J Informetr. (2018) 12(2):481–93. 10.1016/j.joi.2018.03.005

[22] Edwards LC Bryant AS Keegan RJ Morgan K Cooper SM Jones AM. Measuring' Physical literacy and related constructs: a systematic review of empirical findings. Sports Med. (2018) 48(3):659–82. 10.1007/s40279-017-0817-9 29143266 PMC5808047

[23] Guthold R Stevens GA Riley LM Bull FC. Global trends in insufficient physical activity among adolescents: a pooled analysis of 298 population-based surveys with 1.6 million participants. Lancet Child Adolesc Health. (2020) 4(1):23–35. 10.1016/S2352-4642(19)30323-2 31761562 PMC6919336

[24] Hanifah L Nasrulloh N Sufyan DL. Sedentary behavior and lack of physical activity among children in Indonesia. Children. (2023) 10(8):1283. 10.3390/children10081283 37628282 PMC10453900

[25] Chaput JP Willumsen J Bull F Chou R Ekelund U Firth J. 2020 WHO guidelines on physical activity and sedentary behaviour for children and adolescents aged 5–17 years: summary of the evidence. Int J Behav Nutr Phys Act. (2020) 17(1):141. 10.1186/s12966-020-01037-z 33239009 PMC7691077

[26] Sunda M Blazevic I Skugor BG. The relationship between physical literacy and physical fitness in preschool-aged children. Healthcare. (2026) 14(6):708. 10.3390/healthcare14060708 41897162 PMC13026352

[27] Domínguez-Martín G Tárraga-López J López-Gil JF. Relationship between perceived physical literacy and obesity-related outcomes in adolescents: the EHDLA study. Front Public Health. (2024) 12:1321361. 10.3389/fpubh.2024.1321361 38694986 PMC11062133

[28] Clark HJ Dudley D Barratt J Cairney J. Physical literacy predicts the physical activity and sedentary behaviours of youth. Journal of Science and Medicine in Sport. (2022) 25(9):750–4. 10.1016/j.jsams.2022.04.008 35934659

[29] Hsueh MC Chen YH. Associations of physical activity intensity and specific sedentary behaviors during after-school and weekend with overweight and obesity among preschool children. BMC Pediatr. (2025) 25(1):586. 10.1186/s12887-025-05918-9 40751144 PMC12315338

[30] Sport Australia. Australian Physical Literacy Framework. Canberra: Sport Australia (2019).

[31] Tremblay MS Carson V Chaput JP Connor Gorber S Dinh T Duggan M. Canadian 24-Hour Movement guidelines for children and youth: an integration of physical activity, sedentary behaviour, and sleep. Appl Physiol Nutr Metab. (2016) 41(Suppl. 3):S311–27. 10.1139/apnm-2016-0151 27306437

[32] State Council of China. Healthy China 2030 Plan Outline. Beijing: State Council of China (2016).

[33] Eck NJ Waltman L. Visualizing bibliometric networks. In: Ding Y Rousseau R Wolfram D, editors. Measuring Scholarly Impact: Methods and Practice. Cham: Springer International Publishing (2014). p. 285–320.

[34] Donthu N Kumar S Mukherjee D Pandey N Lim WM. How to conduct a bibliometric analysis: an overview and guidelines. J Bus Res. (2021) 133:285–96. 10.1016/j.jbusres.2021.04.070

[35] Caldwell HAT Di Cristofaro NA Cairney J Bray SR MacDonald MJ Timmons BW. Physical literacy, physical activity, and health indicators in school-age children. Int J Environ Res Public Health. (2020) 17(15):5519. 10.3390/ijerph17155367 32722472 PMC7432049

[36] Carl J Barratt J Wanner P Töpfer C Cairney J Pfeifer K. The effectiveness of physical literacy interventions: a systematic review with meta-analysis. Sports Med. (2022) 52(12):2965–99. 10.1007/s40279-022-01738-4 35994237 PMC9691485

[37] Dudley DA. A conceptual model of observed physical literacy. Phys Educ. (2015) 72(5):236–60. 10.18666/TPE-2015-V72-I5-6020

[38] Edwards LC Bryant AS Keegan RJ Morgan K Jones AM. Definitions, foundations and associations of physical literacy: a systematic review. Sports Med. (2017) 47(1):113–26. 10.1007/s40279-016-0560-7 27365029 PMC5215133

[39] Jefferies P Ungar M Aubertin P Kriellaars D. Physical literacy and resilience in children and youth. Front Public Health. (2019) 7:346. 10.3389/fpubh.2019.00346 31803709 PMC6877541

[40] Santos F Newman TJ Aytur S Farias C. Aligning physical literacy with critical positive youth development and student-centered pedagogy: implications for today's Youth. Front Sports Act Living. (2022) 4:845827. 10.3389/fspor.2022.845827 35498526 PMC9039164

[41] Cairney J Dudley D Kwan M Bulten R Kriellaars D. Physical literacy, physical activity and health: toward an evidence-informed conceptual model. Sports Med. (2019) 49(3):371–83. 10.1007/s40279-019-01063-3 30747375

[42] Álvarez-Bueno C Pesce C Cavero-Redondo I Sánchez-López M Garrido-Miguel M Martínez-Vizcaíno V. Academic achievement and physical activity: a meta-analysis. Pediatrics. (2017) 140(6):e20171498. 10.1542/peds.2017-1498 29175972

[43] Longmuir PE Boyer C Lloyd M Yang Y Boiarskaia E Zhu W. The Canadian assessment of physical literacy: methods for children in grades 4 to 6 (8 to 12 years). BMC Public Health. (2015) 15:767. 10.1186/s12889-015-2106-6 26260572 PMC4532252

[44] Fang X Zhang Z. Hotspots and trends in health-oriented physical literacy research: a visual analysis based on the WOS database. BMC Public Health. (2024) 24(1):1480. 10.1186/s12889-024-18951-7 38831413 PMC11145783

[45] Lubans D Richards J Hillman C Faulkner G Beauchamp M Nilsson M. Physical activity for cognitive and mental health in youth: a systematic review of mechanisms. Pediatrics. (2016) 138(3):e20161642. 10.1542/peds.2016-1642 27542849

[46] Grauduszus M. Definitions and assessments of physical literacy among children and youth: a scoping review. BMC Public Health. (2023) 23(1):1746. 10.1186/s12889-023-16680-x 37679785 PMC10486121

[47] Naylor A Keegan R Martin K Flood A. Physical literacy and adult mental health: a cross-sectional survey study. J Sports Sci. (2026) 44(3):335–41. 10.1080/02640414.2025.2578583 41131708

[48] Ryan RM Deci EL. Intrinsic and extrinsic motivation from a self-determination theory perspective: definitions, theory, practices, and future directions. Contemp Educ Psychol. (2020) 61:101860. 10.1016/j.cedpsych.2020.101860

[49] Standage M Ryan RM. Self-Determination theory in sport and exercise. In: Tenenbaum G Eklund RC, editors. Handbook of Sport Psychology. 4th ed., Vol. 1. Hoboken, NJ: John Wiley & Sons (2020). p. 37–56. 10.1002/9781119568124.ch3

[50] Sum RKW Cheng CF Wallhead T Kuo CC Wang FJ Choi SM. Perceived physical literacy instrument for adolescents: a further validation of PPLI. J Exerc Sci Fit. (2018) 16(1):26–31. 10.1016/j.jesf.2018.03.002 30662489 PMC6323161

[51] Rodriguez-Ayllon M Cadenas-Sánchez C Estévez-López F Muñoz NE Mora-Gonzalez J Migueles JH. Role of physical activity and sedentary behavior in the mental health of preschoolers, children and adolescents: a systematic review and meta-analysis. Sports Med. (2019) 49(9):1383–410. 10.1007/s40279-019-01099-5 30993594

[52] Bauman AE Reis RS Sallis JF Wells JC Loos RJF Martin BW. Correlates of physical activity: why are some people physically active and others not? Lancet. (2012) 380(9838):258–71. 10.1016/S0140-6736(12)60735-1 22818938

[53] van Sluijs EMF Ekelund U Crochemore-Silva I Guthold R Ha A Lubans D. Physical activity behaviours in adolescence: current evidence and opportunities for intervention. Lancet. (2021) 398(10298):429–42. 10.1016/S0140-6736(21)01259-9 34302767 PMC7612669

[54] Barnett LM Jerebine A Keegan R Watson-Mackie K Arundell L Ridgers ND. Validity, reliability, and feasibility of physical literacy assessments designed for school children: a systematic review. Sports Med. (2023) 53(10):1905–29. 10.1007/s40279-023-01867-4 37341907 PMC10504218

[55] Telama R Yang X Leskinen E Kankaanpää A Hirvensalo M Tammelin T. Tracking of physical activity from early childhood through youth into adulthood. Med Sci Sports Exerc. (2014) 46(5):955–62. 10.1249/MSS.0000000000000181 24121247

[56] Hulteen RM Barnett LM True L Lander NJ Del Pozo Cruz B Lonsdale C. Validity and reliability evidence for motor competence assessments in children and adolescents: a systematic review. J Sports Sci. (2020) 38(15):1717–98. 10.1080/02640414.2020.1756674 32608334

